# Feedback Loops of the Mammalian Circadian Clock Constitute Repressilator

**DOI:** 10.1371/journal.pcbi.1005266

**Published:** 2016-12-12

**Authors:** J. Patrick Pett, Anja Korenčič, Felix Wesener, Achim Kramer, Hanspeter Herzel

**Affiliations:** 1 Institute for Theoretical Biology, Humboldt-Universität zu Berlin, Berlin, Germany; 2 Center for Functional Genomics and Biochips, Institute of Biochemistry, Faculty of Medicine, University of Ljubljana, Ljubljana, Slovenia; 3 Laboratory of Chronobiology, Charité-Universitätsmedizin Berlin, Berlin, Germany; 4 Institute for Theoretical Biology, Charité-Universitätsmedizin Berlin, Berlin, Germany; Ecole Normale Supérieure, FRANCE

## Abstract

Mammals evolved an endogenous timing system to coordinate their physiology and behaviour to the 24h period of the solar day. While it is well accepted that circadian rhythms are generated by intracellular transcriptional feedback loops, it is still debated which network motifs are necessary and sufficient for generating self-sustained oscillations. Here, we systematically explore a data-based circadian oscillator model with multiple negative and positive feedback loops and identify a series of three subsequent inhibitions known as “repressilator” as a core element of the mammalian circadian oscillator. The central role of the repressilator motif is consistent with time-resolved ChIP-seq experiments of circadian clock transcription factors and loss of rhythmicity in core clock gene knockouts.

## Author Summary

Circadian clocks are endogenous oscillators that drive daily rhythms in physiology, metabolism and behavior. The recent years have witnessed enormous progress in our understanding of the mechanistic and genetic basis of these clocks. While mathematical modelling has made important contributions to our current view of the circadian clock network, it is still debated, which network motifs are necessary and sufficient for generating self-sustained oscillations. Exploiting a data-driven mathematical model we here identify the “repressilator” as a key design principal. The central role of the repressilator motif is consistent with time-resolved binding data of circadian clock transcription factors and loss of rhythmicity in core clock gene knockouts.

## Introduction

An autonomous circadian clock controls daily rhythms in physiology and behaviour in a large variety of species. Such an endogenous timing system has evolved to adapt to the 24h period of the solar day. Circadian rhythms are generated by intracellular transcriptional feedback loops involving cis-regulatory elements such as E-boxes, D-boxes, and ROR-elements (RREs). In mammals, more than 20 core clock genes assemble a sophisticated gene regulatory network with multiple negative and positive feedback loops [1]. Given the complexity of this network, we here investigate, which network motifs are necessary and sufficient for generating self-sustained rhythms.

The classical view of the circadian oscillator considers the E-box mediated negative feedback of Period (PER) and Cryptochrome (CRY) proteins towards the transcriptional activator complex CLOCK/BMAL1 as the major driver of circadian rhythms [2]. More recent studies also suggest that another negative feedback loop with the nuclear receptors *Rev-erb-α* and *Rev-erb-β* acting through RORE enhancers is not merely an auxiliary loop, but is capable of generating self-sustained oscillations [3, 4]. Indeed, double-knockouts of *Rev-Erb* genes destroy rhythmicity [5, 6]. The relative importance of clock genes and their regulatory interactions is consequently debated [7].

Here, we explore which gene regulatory motifs are most relevant for 24h oscillations. To this end, we systematically analyzed a recently published circadian oscillator model [8]. This model includes *Bmal1* as a driver of E-box mediated transcription, *Per2* and *Cry1* as early and late E-box repressors, respectively, as well as the D-box regulator Dbp and the nuclear receptor *Rev-erb-α*. The model design is based on experimentally verified regulatory interactions, degradation rates and post-transcriptional delays. The unknown parameters describing transcriptional regulation have been fitted to four qPCR data sets using an evolutionary optimization algorithm [8]. The resulting gene network involves 17 regulatory interactions forming multiple negative and positive feedback loops and therefore contains several potential oscillation generating mechanisms.

Such a quantitative model is well suited to study the principles of circadian rhythm generation. We comprehensively and systematically analyze the capability of sub-networks to generate oscillations. Interestingly, we identify the “repressilator” motif [9–12] as a central loop of the mammalian circadian oscillator. The repressilator comprises a series of three inhibitions including the genes whose knockouts lead to arrhythmicity, i.e. *Cry*, *Per* and *Rev-erb*.

## Results

### A 5-gene model represents the core oscillator

To study the complex gene regulatory network of the mammalian circadian oscillator, we constructed a mathematical model with only the key components as explicit variables. For example, transcriptional profiles reveal clear redundancies in the network of core clock genes [1, 4] with RORE-binding activators (*Rorα,-β,-γ*) exhibiting opposite phases as the RORE-binding inhibitors (*Rev-erb-α,-β*). This allows to describe the regulatory actions by a single term controlled by *Rev-erb-α* levels, while the systems behaviour remains the same. The additional effects by *Ror*-genes and *Rev-erb-β* can be taken into account by changes of parameters describing the strength of *Rev-erb-α* regulation. Analogously, we combine the regulations via D-boxes into one term. The *Dbp*-gene represents the combined effects of the activators *Dbp*, *Hlf* and *Tef* and the inhibitor *E4bp4*. Transcriptional regulation via E-boxes is particularly complex [13]. In our model, *Bmal1* quantifies the positive regulation after dimerization with *Clock* or *Npas2*, while the genes *Per2* and *Cry1* represent early and late E-box driven genes, respectively. The essential role of a rather late *Cry1* phase has been demonstrated in detail elsewhere [14, 15].

**Fig 1:**
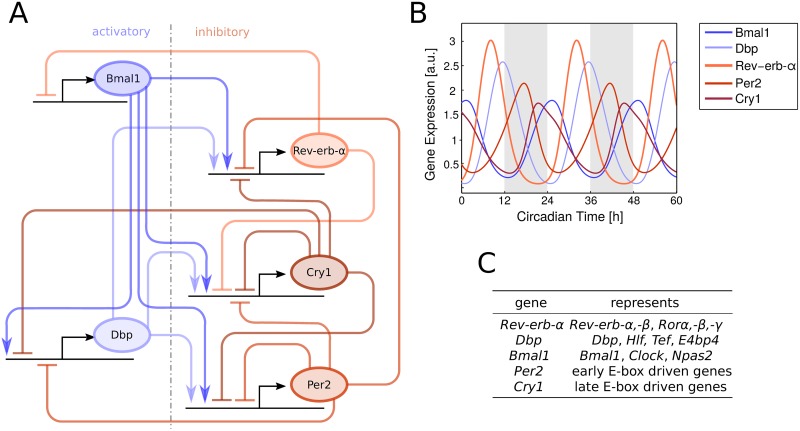
(A) Network graph of the circadian oscillator model. Activating and inhibiting influences between genes are colored in blue and red, respectively. (B) Simulation of gene expression of all 5 genes. (C) Each variable in the model represents a group of genes with similar functional characteristics.

Overall, we designed a regulatory network consisting of five variables only [8]. Fig 1A shows that even this core clock network exhibits multiple negative and positive feedback loops. Importantly, our model successfully describes published phase relations, amplitudes and waveforms of clock gene expression profiles (Fig 1B). A detailed comparison with experimentally measured profiles is described in [8].

Our gene regulatory network model contains 34 kinetic parameters. Since quantitative details of post-transcriptional processes including phosphorylations, complex formations and nuclear translocation are not known, we represent these processes by explicit delays taken from experimentally determined phase-differences between peak expression of mRNA and protein [4]. Degradation rates were taken from large scale studies of mRNA decay [16, 17] and protein measurements [18–20]. Exponents in transcriptional regulation terms are derived from the number of experimentally verified cis-regulatory elements [4, 21].

The remaining parameters describe the kinetics of transcriptional regulation, which is not known in quantitative detail. Thus, we applied global optimization techniques to fit parameters to carefully normalized qPCR data sets from mouse liver and adrenal gland [8]. For both tissues data from light-dark cycles (LD) and constant darkness (DD) were available. Interestingly, all four expression profiles show clear similarities and thus we fitted a consensus model to these four data sets. The complete set of equations and parameters is provided in (S1 Appendix).

The resulting data-driven gene regulatory network model allows to address the following questions: (i) Which kinetic parameters are most relevant for 24h rhythm generation? (ii) Are oscillations of all five genes necessary for self-sustained rhythms? (iii) What are the most essential regulatory interactions required for rhythm generation? We will answer these questions in the next sections by systematically varying parameters and clamping gene expression levels to their mean values. Thereby, we identify design principles in the network necessary and sufficient for generating circadian oscillations.

### Period jumps upon parameter variations suggest coexisting oscillators

**Fig 2:**
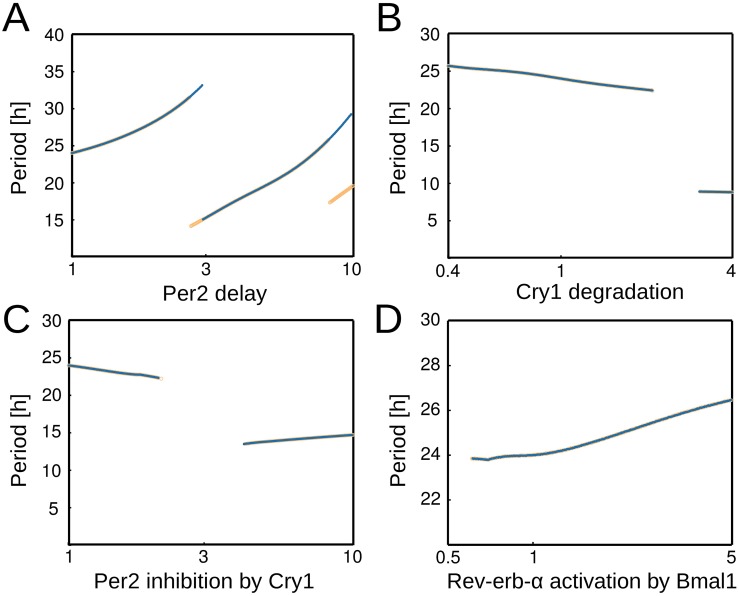
Effect of parameter alterations on the period (fraction of default value on logarithmic x-scale). (A) Change of *Per2* delay. (B) Change of *Cry1* mRNA degradation rate. (C) Change of Cry1 inhibition strength on Per2. (D) Change of Bmal1 activation strength on *Rev-erb-α*. The default parameter values, corresponding to 1 on the x-axis, are: *Per2* delay *τ*_3_ = 3.82, *Cry1* degradation *d*_4_ = 0.2, *Rev-erb-α* activation by *Bmal1* *actn*_1,2_ = 3.26 and *Per2* inhibition by *Cry1* *inh*_4,3_ = 0.37. Blue symbols refer to increasing parameters, whereas orange symbols refer to the reverse parameter variation (see S2 Appendix for details).

Our set of default parameters has been fitted to mRNA expression profiles of circadian clock genes from mouse liver and adrenal gland. It is conceivable that the chosen kinetic parameters are different among tissues and also depend on the specific physiological conditions. In order to test which parameters are most relevant for 24h oscillations, we varied all parameters by two orders of magnitude around the default values. Fig 2 represents the results for four particularly interesting parameters. The periods are plotted for parameter values where self-sustained oscillations occur.

It turns out that oscillations persist for wide ranges of kinetic parameters supporting the robustness of the model. The period increases with the delay between *Per2* transcription and its function as an inhibitor (Fig 2A). Indeed, the FASPS mutation of PER2 reduces protein life-time, leading to a faster turn-over and hence to shorter delays [22], thereby implying a shorter period and much earlier phases [23]. Increasing the degradation rate of *Cry1* mRNA leads to period shortening as expected (Fig 2B) and consistent with the shorter period of the *Cry1*^-/-^ knockout mice [24].

In addition, appropriate interactions of *Bmal1*, *Rev-erb-α*, *Per2* and *Cry1* are required to generate self-sustained rhythms (Fig 2C and 2D). Variations of kinetic parameters associated with transcriptional regulations have minor effects on the period near their default values, consistent with the observation that the clock is resilient to changing transcription rates [25].

The most surprising observation, however, are period jumps for somewhat larger parameter changes (Fig 2). In particular, the detection of long and short periods within a very narrow parameter range suggests that multiple mechanisms might co-exist which can generate self-sustained rhythms. Indeed, the systematic analysis described below allows us to attribute oscillations with different period to specific loops in the model.

For example upon increasing the *Per2* delay, the period falls down to 15h after rising up to more than 30h (Fig 2A). Here, the period jump occurs, when the explicit delay is very large (above 10.5h) and influences a subsequent cycle of the oscillations rather than the current one. The rhythm-generating loop, however, remains the same. Further increase of the delay then leads again to an increase of the period until the phase pattern and period before the jump emerges again. An animation of this gradual parameter variation is provided as S1 Video. Interstingly, there is hysteresis near the period jumps, indicating coexisting limit cycles (also termed “birhythmicity” [26]).

A period jump also occurs upon variation of the *Cry1* degradation rate (Fig 2B). Here, the jump to a short period of < 10h is associated with a change of the rhythm-generating loop: *Cry1* self-inhibition generates these oscillations. Since the self-inhibitory loop exhibits a rather short delay of *τ*_*Cry*1_ = 3.13h, the resulting period is consequently quite small (comprehensive list of feedback loops and delays in the S4 Appendix). We show in S2 Appendix that in the transition region two rhythms persist (termed “torus”).

If we increase *Per2* inhibition by *Cry1*, oscillations vanish via a supercritical Hopf bifurcation. At much larger parameter values another Hopf bifurcation leads to a limit cycle governed by a double-negative feedback loop involving *Per2*, *Dbp* and *Cry1*.

Taken together, even a relatively small network of just five genes can establish multiple mechanisms generating oscillations, some with periods in the circadian range. While it is generally believed [2] that the negative feedback loop via *Per*/*Cry* is the primary driver of circadian oscillations, these multiple regulatory mechanisms even within a relatively small network raise the possibility that the underlying key mechanism for circadian rhythm generation is more complex.

### Several sub-networks can generate oscillations

To investigate, which network nodes (genes) are essential for circadian rhythm generation we systematically studied all possible sub-networks under default parametrization.

Our gene network with 7 positive and 10 negative regulations exhibits multiple feedback loops (Fig 1A). Delayed negative feedback loops constitute the basic elements of self-sustained oscillations [27, 28]. Often these negative feedback loops are interlinked with positive feedbacks ensuring robust and tunable rhythms [29–33]. Thus, we focused on which sub-networks forming feedback loops are able to generate sustained rhythms for physiologically plausible parameters.

To this end, we systematically clamped all possible combinations of gene-subsets to their respective oscillation mean values. The mean values are obtained from simulations of the complete network. Clamping the level of *Dbp* or *Bmal1*, for example, retains the corresponding positive regulations, but excludes *Dbp* and *Bmal1* as drivers or transmitters of oscillations, thereby focusing on the remaining genes. The clamping to mean values ensures that the system remains near the carefully tuned and physiologically reasonable default configuration. Clamping of genes corresponds to constitutively expressed genes using non-rhythmic promoter constructs [15, 34–36]. Compared to knockout studies, our clamping protocol is less invasive and keeps the system close to its physiological ranges.

**Fig 3:**
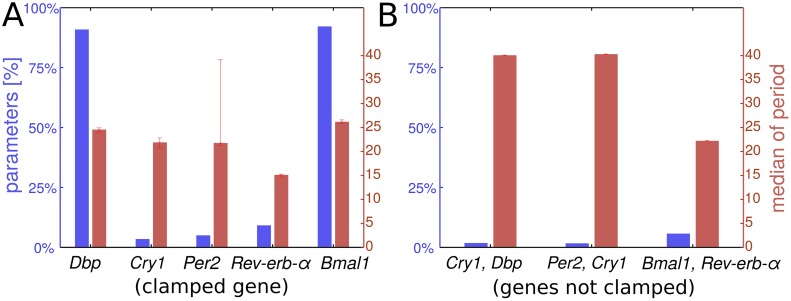
*Cry1*, *Per2* and *Rev-erb-α* oscillations are most critical for circadian rhythm generation. All possible combinations of gene-subsets were analyzed for oscillating solutions by clamping the remaining genes to their respective oscillation mean values (A: one gene clamped; B: three genes clamped). Blue bars indicate the percentage of parameter sets around the default values that result in oscillating solutions. Red bars depict the median period among these solutions. Only 3 of 10 combinations of 2 genes oscillate at all, which are shown in (B). Error bars give the upper and lower quartiles for the period.

There are 5 combinations of 4 genes resulting from clamping only one single gene. For all of the resulting networks there exist certain parameter configurations with oscillatory solutions (blue bars in Fig 3). Clamping *Rev-erb-α*, *Per2* or *Cry1* has strong effects: Using the default parameters of the complete network, oscillations vanish. In order to explore the rhythm-generating capabilities of the sub-networks more extensively around the default parameter set, we varied each parameter of the system in a range from 5-fold reduction to 5-fold increase in repeated simulations with 200 points on a log scale. For every simulation we tested, whether or not the sub-network oscillates (Fig 3A). It turns out that in principle all sub-systems of 4 genes are capable of generating oscillations with reasonable periods.

Interestingly, clamping *Dbp* (first blue bar, Fig 3A) or *Bmal1* (last blue bar, Fig 3A) sustains oscillations in about 90% of parameter combinations with a median period close to 24h. This is in line with experimental findings showing that *Bmal1* cycling is not necessary for circadian rhythms [36, 37], *Dbp*^-/-^ knock-out mice are still rhythmic [38] and triple-knockouts of D-box regulators have only minor effects [39]. Thus, both experimental evidence and our modelling results underline that *Bmal1* and *Dbp* cycling is not essential for sustaining oscillations.

**Fig 4:**
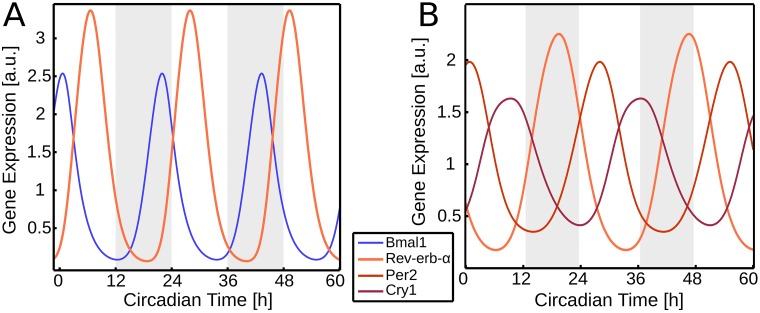
Oscillations of sub-networks. (A) Simulation of gene expression of *Rev-erb-α* and *Bmal1* with other genes (*Cry1*, *Per2* and *Dbp*) clamped to their constant mean value. Upon doubling the strength of *Bmal1* to *Rev-erb-α* activation, oscillations are rescued with a period of 24h. (B) Simulation of gene expression of *Rev-erb-α*, *Per2* and *Cry1*, with other genes (*Dbp* and *Bmal1*) clamped to their constant mean value. The period lengthens, but oscillations are retained without parameter adjustments being necessary.

Simultaneously clamping two genes to their mean values results in $\left(\begin{array}{c} 5\\ 3 \end{array}\right)=10$ sub-networks of 3 genes. We find that 5 of these networks are capable of generating self-sustained oscillations, when allowing up to 5-fold adjustments of single parameters. Interestingly, *Rev-erb-α* is present in most of these oscillatory sub-systems (as an example, see Fig 4B).

Simultaneously clamping 3 genes leads to $\left(\begin{array}{c} 5\\ 2 \end{array}\right)=10$ sets of only 2 remaining genes. Surprisingly, 3 of these pairs are still able to oscillate for appropriate parameter adjustments (Fig 3B). Notably, the negative feedback loop involving *Bmal1* and *Rev-erb-α* oscillates with a period of about 24 hours after only a minor parameter change (compare Fig 4A). It turns out that some of the previously identified oscillations in larger sets of 3 and 4 genes can be traced back to this simple loop. This finding confirms earlier observations that the feedback loop via nuclear receptors can serve as a possible mechanism for rhythm generation [3, 4, 7].

### The repressilator is the most essential regulatory motif

In the previous section gene levels (nodes) were clamped to their mean values, allowing sub-networks to be identified as possible rhythm generators. Now we expand our approach to combinatorial clamping of regulatory interactions (edges in the network graph in Fig 1A) allowing the identification of sub-networks on a process-level. Thereby, network motifs most essential for the generation of 24h rhythms can be identified.

In our model, transcriptional regulations are described by products of activating and inhibiting terms corresponding to the influence of regulating genes [4]. If the expression value of a regulating gene is set constant to its mean value in the term of only one specific target-gene, we call the corresponding interaction “clamped”. For more details on the method, see (S3 Appendix).

Since the gene network contains altogether 17 regulatory interactions, there are 2^17^ = 131,072 combinations, or ON/OFF configurations, if OFF means clamping. For all these combinations we tested in detail, whether or not oscillations persist, but did not consider additional variation of kinetic parameters. We found that 14,125 (about 10%) of all network configurations oscillate.

In order to evaluate the importance of specific regulatory interactions we calculated for each interaction the relative frequency of inclusion in the set of oscillatory network configurations. Among all possible configurations any given process is ON or OFF in 50% of the cases. Thus, considering the set of oscillatory ON/OFF configurations, an edge that is not part of the essential loop would still occur in one-half of the cases.

Indeed, the analysis of all oscillatory ON/OFF configurations reveals that most of the processes occur in 50% of the oscillating configurations as expected for a non-essential process. However, a distinct set of regulatory interactions turned out to be present in almost 100% of the oscillating network configurations.

**Fig 5:**
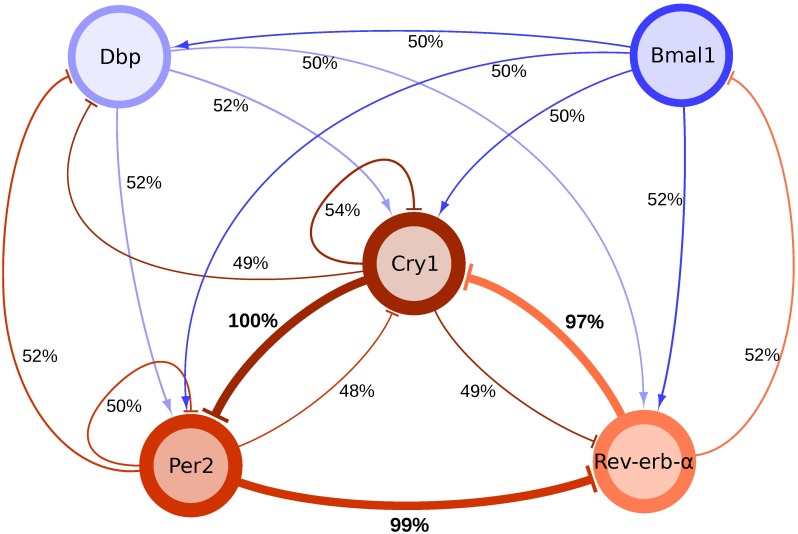
The repressilator comprising *RevErba*, *Per2* and *Cry1*. The relative abundancy of processes in oscillating sub-networks is mapped to the edge width. All edges of the repressilator are highly prominent among all oscillating networks, which reflects its role as the dominant source of oscillations in the model.

To our surprise, only 3 of the 17 regulatory interactions are exceptionally important to keep the network rhythmic, occurring in almost every oscillating configuration (marked in Fig 5 by thick red lines). All other regulations can be clamped to prevent them from transmitting rhythms: The remaining 3 regulatory interactions still retain oscillations. While the period generated by this 3-gene sub-network in isolation is somewhat longer upon default parametrization, the full network compensates this by fine-tuning through other regulations, including a feedforward loop [40]. Interestingly, the three regulations are all inhibitory: *Per2* inhibits *Rev-erb-α*, *Rev-erb-α* inhibits *Cry1* and *Cry1* inhibits *Per2*.

Such a symmetric triangular motif of inhibitory interactions has been introduced as a paradigm of synthetic oscillators termed “repressilator” [9].

### The repressilator motif is a robust oscillator

In contrast to most models of the circadian clock, which are essentially based on variations of the Goodwin model [22, 41], the repressilator comprises three subsequent inhibitions rather than a single negative feedback. It is known that classical Goodwin-based models need strong negative cooperativity (minimal Hill coefficient of 8—probably unrealistic biochemically) and long balanced degradation times to obtain self-sustained oscillations [28, 33, 42]. Within the repressilator, however, the delay and the required non-linearities can be distributed over the three inhibitions.

To test the compatibility of the repressilator, we performed a robustness analysis of two simple prototypic models with a single feedback loop, one with only one inhibition and one with the repressilator motif (for details see S5 Appendix). In particular, we generated random parameter sets for both models and compared the frequencies of self-sustained oscillations and the minimal Hill coefficients necessary to generate oscillations (see Fig. 3 in S5 Appendix). We found that the repressilator model has a higher fraction of oscillations and can oscillate with fairly low Hill coefficients of about 2. Note, that modified Goodwin oscillators with additional nonlinearities allow reductions of the Hill coefficient as well [43, 44]. Generally, systems with multiple nonlinearities and delayed feedbacks allow robust oscillations with reasonable Hill coefficients [32, 45, 46]. The repressilator motif allows to distribute nonlinearities and delays.

## Discussion

About 20 years ago the first mammalian core clock genes including CLOCK, BMAL1, PERIODs and CRYs were discovered [2]. Double knock-outs of PER and CRY lead to arrhythmic behaviour in mice [24, 47]. Consequently, the first models of the mammalian circadian clock considered the negative feedback loop via PER/CRY inhibition as the central element for rhythm generation [48–50], whereas the REV-ERB loop [19] was considered merely as an auxiliary loop. In 2011, using a comprehensive modelling approach we proposed that both loops, the PER/CRY feedback and the REV-ERB feedback, can generate circadian oscillations [3], and indeed, one year later experimental findings that *Rev-erb-α* and *Rev-erb-β* lead to arrhythmicity [5, 6] confirmed our model.

The 5-gene model studied here contains both loops [8], allowing us to systematically and comprehensively explore the rhythm generating mechanisms in this system. We were surprised to detect large period jumps upon certain parameter variations (see Fig 2) indicating that multiple feedback oscillators might be embedded in the regulatory network.

The observation of multiple mechanisms to generate oscillations raises the question whether or not multiple rhythms have been described experimentally. Indeed, for knockouts and specific light conditions, “splitting” has been found [51–53]. In most cases, these multiple rhythms are assigned to heterogeneity within the circadian pacemaker, the suprachiasmatic nucleus [54, 55]. To our knowledge, there is no experimental evidence of frequency jumps in isolated cells or tissues. However, in some cases *Bmal1* and *Per2*-reporters indicate slightly different periods [56], indicating perhaps different mechanisms to generate oscillations.

By clamping genes to their mean values we identified several sub-networks as potent rhythm generators (see Figs 3 and 4). As described in S2 Appendix, these sub-networks include *Cry1* self-inhibitions and double-negative feedback loops discussed earlier as a robust design principle [32]. Interestingly, many of these sub-networks include the PER/CRY and REV-ERB loops discussed above.

In order to identify the design motif most essential for circadian rhythm generation, we systematically clamped all 17 regulations of our network in all possible configurations (i.e. 131,072) and found that about 10% were rhythmic. Reverse-engineering of these 14,125 rhythmic configurations uncovered the key finding of our study: The most essential regulations form a well-studied motif—the repressilator [9]—which is key for more than 97% network configurations (Fig 5). Most interestingly, this repressilator motif involves elements of both negative loops discussed as distinct mechanisms previously [3].

A repressilator sub-network was suggested earlier in the context of the mammalian clock [11], however, with different nodes and edges involving E-boxes, D-boxes and RREs. In contrast, our study identifies two inhibitors of the E-box, *Per2* and *Cry1* as a part of the repressilator. Thus, both the inhibition of *Per2* by *Cry1* via E-boxes and the inhibition of *Rev-erb-α* by *Per2* via E-boxes appear to play a major role.

A repressilator was also suggested for the plant circadian clock [10, 57] supporting—despite the difference of the mammalian and plant clock—the hypothesis that a rhythmic gene regulatory network using a repressilator motif can generate circadian rhythms.

In most previous models of the mammalian clock *Per* and *Cry* act through the PER/CRY-complex in a symmetrical way. Recent experimental studies [13, 15, 58] stressed the essential role of delayed *Cry1* expression and DNA binding. Our data-based model includes the late phase of *Cry1*. Thus, the sequential inhibitions by *Rev-erb*, *Per* and *Cry1* can generate sustained oscillations. In order to illustrate that the repressilator motif is not restricted to our specific model based on liver and adrenal gland data, we fitted another 5 gene model to recent data of kidney expression profiles [59, 60]. It turned out, that clamping all regulations except for the repressilator motif kept the oscillations going with comparable period and amplitudes (S7 Appendix).

### Agreement with experimental results

The repressilator motif is represented as a serial inhibition of *Cry1* via *Rev-erb-α*, of *Rev-erb-α* via *Per2* and of *Per2* via *Cry1*. The two activators *Bmal1* and *Dbp* can be clamped to their mean values without loosing oscillations.

It has indeed been reported that constant *Bmal1* levels can sustain rhythms [36, 37] and that triple-knockouts of D-box regulators have only minor effects on circadian rhythmicity [39]. In contrast, double-knockouts of *Cry*, *Per* and *Rev-erb* genes lead to arrhythmicity [5, 24, 47] supporting our finding that circadian rhythms are not generated by a single negative feedback loop, but by a gene regulatory network with a repressilator as a core motif.

Double-knockouts induce behavioral arrhythmicity. Since core clock genes oscillate in surprisingly similar phase relationships in almost all tissues [8, 59, 61], it is very likely that the KO experiments imply also tissue arrythmicity. Indeed, studies of double-knockouts include data on arrhythmic tissues and cells [5, 47, 62].

In previous studies, models have been adapted to available mutant phenotypes [3, 32, 63]. Since our variables group together genes with similar regulatory effects, a comparison with knockout data is not easy. Our clamping protocols resemble constitutive expression or overexpression, and thus we discuss related experiments. It has been shown that constitutive expression or overexpression of *Per* genes impairs rhythms [34, 35, 64, 65]. Similarly, constitutive or out-of-phase expression of *Cry1* impairs rhythmicity [15] and overexpression of *Cry1* leads to arrhythmicity [58]. Knockouts and knockdowns of *Cry1* lead to arrhythmicity in tissues and cells [62, 66], even though the coupling within the SCN can rescue rhythmicity [62] corresponding to a short-period phenotype of *Cry1* knockouts [24]. Interestingly, knockouts and knockdowns of *Cry2*, an early E-box target not regulated by *Rev-erb-α*, stay rhythmic with large amplitudes [62, 66, 67]. The essential role of *Rev-erb-α* inhibition of *Cry1* is demonstrated by the removal of the intronic ROR-elements leading to early phases of *Cry1* and vanishing amplitudes in single cells [14]. In summary, there is strong experimental evidence that the cycling of the 3 repressilator genes is of central importance for a cellular clock.

Our 5-gene model is based on carefully normalized qPCR data of liver and adrenal gland [8]. More recently, expression profiles of 14 different tissues have been published [59]. In all of these tissues the repressilator genes are oscillating with significant amplitudes and with serially ordered phases consistent with the repressilator mechanism (see S6 Appendix). Similar observations were reported by Yamamoto et al. [61].

In addition to mRNA rhythms protein oscillations are relevant to understand regulatory processes. Unfortunately, liver proteome studies could not quantify core clock protein rhythms due to limited resolution [68, 69]. A recent quantification of clock proteins confirms early protein expression of REV-ERB*α*, followed by peaks of PER2 and CRY1 [70]. Recent ChIP-Seq experiments allow the estimation of binding phases of regulatory clock proteins [5, 6, 13, 71]. It turns out that REV-ERB*α* binds early (Circadian Time CT = 6–10), followed by PER2 binding around CT16 and CRY1 binding at around CT24. These subsequent binding peaks are fully consistent with the proposed repressilator mechanism.

### Synergy of feedback regulations

Our starting point was a gene-regulatory model based on expression profiles of core clock genes in mouse liver and adrenal gland. As shown in Fig 5, the repressilator is the dominant motif of this gene-regulatory network.

However, Figs 3 and 4 illustrate that also other negative feedback loops are capable of generating oscillations. Furthermore, positive feedback loops are known to support rhythm generation [33]. A comprehensive list of loops within our gene regulatory network is given in (S4 Appendix), showing the interrelations and coherence of loops. Our results suggest, that multiple loops support the generation of circadian oscillations, while the repressilator constitutes an essential core mechanism: While the pure repressilator generates oscillations with increased periods, the addition of other regulations including a feedforward loop [40] from *Cry1* to *Per2* via *Dbp* tune the period to values of about 24h.

In summary, our comprehensive analysis of a data-driven core-clock model suggests that the synergy of multiple regulatory motifs allows robust and tunable self-sustained oscillations. We further propose, that a series of subsequent inhibitions known as repressilator constitutes a core motif of the mammalian circadian clock gene-regulatory network.

## Supporting Information

S1 Appendix DDE equations. Equations of the underlying mathematical model.
(PDF)

S2 Appendix Period jumps. Details on period jumps observed for specific parameter variations.
(PDF)

S3 Appendix Combinatorial analysis of sub-models. Details on the clamping procedure extensively used in this work.
(PDF)

S4 Appendix Feedback- and feedforward loops. Overview and analysis of the relation of loops in the core clock network.
(PDF)

S5 Appendix Robustness comparison. Details on the robustness comparison of repressilator and Goodwin oscillator described in the results section.
(PDF)

S6 Appendix Phases of repressilator genes in additional experimental data. mRNA expression phases in different tissues.
(PDF)

S7 Appendix Model in kidney. Repressilator in the same model structure fitted to kidney expression data.
(PDF)

S1 Video Simulation of parameter variation. Increasing and collapsing period upon increase of *Per2* delay.
(MP4)

S2 Video Simulation of parameter variation. Phase portrait showing a torus upon increase of *Cry1* degradation rate.
(MP4)
