# Supplementary material for: Feedback Loops of the Mammalian Circadian Clock Constitute Repressilator
Source: PLoS Comput Biol. 2016 Dec 12;12(12):e1005266. doi: 10.1371/journal.pcbi.1005266 (PMC5189953; doi:10.1371/journal.pcbi.1005266)
Supplement: S1 Appendix — Equations of the underlying mathematical model. (PDF) [file pcbi.1005266.s001.pdf]

S1 Underlying mathematical model

The Delay-Differential Equation core clock model

The mammalian circadian clock model used in this paper was published in 2014 by (Korenčič et al., 2014). It is a condensed representation of the core clock in mammals, since effectively similar parts such as, for example, *Rev-erb-*α and -β mRNA expression time courses were represented by a single variable. Furthermore, the underlying Delay-Differential Equations (DDE) introduce explicit delays to the model, which represent a series of biological events that are not known in quantitative detail, but contribute to a marked time-delay between cause and effect.

As a result of condensing the current knowledge on core clock regulation, a relatively small network of 5 nodes was obtained, which yet comprises a large number of processes. It is conveniently abstract, but still allows us to fully distinguish candidate mechanisms relevant for the generation of circadian rhythms (also see S4 Appendix). We extensively tested the robustness of the model regarding parameter variations (see Supporting Information in Korenčič et al. (2014)). It turned out, that oscillations were fairly robust (see also Figure 2 regarding 4 specific parameters). Thus, this data-driven model constitutes an ideal starting point for the focused analysis conducted in this work.

We here summarize the main features of the model. For a more detailed description, please refer to the original publication.

Experimental data

Model topology was constructed from a comprehensive survey of known interactions, usually identified via ChIP-Seq experiments and mapping of transcription factor binding motifs. Time-resolved mRNA expression profiles were used to fit parameters of the model. For degradation rates and the delay parameters, values taken from the literature were used to constrain the fitting procedure.

Mathematical representation

The Delay-Differential Equations use a constant delay for each gene to describe the time difference between reaching a particular expression level and its effect on other Differential Equations. Thus, five delay parameters named $\tau_i, i \in 1,\ldots,5$ exist and are used to access past expression levels, i.e. at time $t - \tau_i$. Expression levels are denoted by $[gene_i]$, where the gene name is enclosed in square brackets. The genes are numbered as: 1–$Bmal1$, 2–$RevErba$, 3–$Per2$, 4–$Cry1$, 5–Dbp.

The equation of each of the five genes is composed of different modulator terms representing activations or inhibitions. Each modulator term corresponds to an edge in the network graph. In the end of each equation is a degradation term with degradation rate $d_i, i \in 1,\ldots,5$.

Inhibitory modulators have the form:

$$\left(\frac{1}{\frac{[\mathrm{gene_j}](\mathrm{t}-\tau_\mathrm{j})}{\mathrm{inh_{j,i}}}+1}\right)^n \qquad \textbf{(S1-1)}$$

where the $gene_i$, which the corresponding equation belongs to, is inhibited by $gene_j$. The strength of the inhibition is set via the kinetic parameter $inh_{j,i}$. The number of experimentally verified transcription factor binding sites is accounted by n.

There is no direct experimental evidence that the exponents equal the number of verified cis-regulatory elements. We follow previous studies, assuming that multiple binding sites enhance cooperativity (Höfer et al. (2002); Bintu et al. (2005)). There is experimental evidence that the inhibition of *Cry1* via *Rev-erb-*α is quite strong (Ukai-Tadenuma et al. (2011)). Furthermore, *Per2* and *Rev-erb-*α expressions are driven by multiple E-boxes (summarized in Korenčič et al. (2012), Supporting Information S4.1) supporting strong inhibitions. These findings make a choice of exponents 2 and 3 reasonable.

Activatory modulators have the form:

$$\left(\frac{\frac{\mathrm{actn_{j,i}}\cdot[\mathrm{gene_j}](\mathrm{t}-\tau_\mathrm{j})}{\mathrm{act_{j,i}}}+1}{\frac{[\mathrm{gene_j}](\mathrm{t}-\tau_\mathrm{j})}{\mathrm{act_{j,i}}}+1}\right)^n \qquad \textbf{(S1-2)}$$

where $gene_i$ is again the target, and $gene_j$ is the activating gene. The strength of activation is set via both of the kinetic parameters $act_{j,i}$ and $actn_{j,i}$. Similar to inhibitory modulators, the number of transcription factor binding sites is represented in the power n.

Full set of equations

The full set of DDEs is given in Equation **S1-3** below.

Equation S1-3 Full set of Delay-Differential Equations (DDEs) of the circadian core clock model.

$$\frac{d\,[Bmal1]}{dt} = \left(\frac{1}{\frac{[\mathrm{RevErb}_\alpha](t-\tau_2)}{\mathrm{inh}_{2,1}}+1}\right)^2 - \mathrm{d1}\,[Bmal1]\,(t)$$

$$\frac{d\,[RevErb_\alpha]}{dt} = \left(\frac{\frac{\mathrm{actn}_{1,2}\,[\mathrm{Bmal1}](t-\tau_1)}{\mathrm{act}_{1,2}}+1}{\frac{[\mathrm{Bmal1}](t-\tau_1)}{\mathrm{act}_{1,2}}+1}\right)^3 \left(\frac{1}{\frac{[\mathrm{Per2}](t-\tau_3)}{\mathrm{inh}_{3,2}}+1}\right)^3 \left(\frac{\frac{\mathrm{actn}_{5,2}\,[\mathrm{Dbp}](t-\tau_5)}{\mathrm{act}_{5,2}}+1}{\frac{[\mathrm{Dbp}](t-\tau_5)}{\mathrm{act}_{5,2}}+1}\right)\left(\frac{1}{\frac{[\mathrm{Cry1}](t-\tau_4)}{\mathrm{inh}_{4,2}}+1}\right)^3 - \mathrm{d2}\,[RevErb_\alpha]\,(t)$$

$$\frac{d\,[Per2]}{dt} = \left(\frac{\frac{\mathrm{actn}_{1,3}\,[\mathrm{Bmal1}](t-\tau_1)}{\mathrm{act}_{1,3}}+1}{\frac{[\mathrm{Bmal1}](t-\tau_1)}{\mathrm{act}_{1,3}}+1}\right)^2 \left(\frac{1}{\frac{[\mathrm{Per2}](t-\tau_3)}{\mathrm{inh}_{3,3}}+1}\right)^2 \left(\frac{\frac{\mathrm{actn}_{5,3}\,[\mathrm{Dbp}](t-\tau_5)}{\mathrm{act}_{5,3}}+1}{\frac{[\mathrm{Dbp}](t-\tau_5)}{\mathrm{act}_{5,3}}+1}\right)\left(\frac{1}{\frac{[\mathrm{Cry1}](t-\tau_4)}{\mathrm{inh}_{4,3}}+1}\right)^2 - \mathrm{d3}\,[Per2]\,(t)$$

$$\frac{d\,[Cry1]}{dt} = \left(\frac{1}{\frac{[\mathrm{RevErb}_\alpha](t-\tau_2)}{\mathrm{inh}_{2,4}}+1}\right)^2 \left(\frac{\frac{\mathrm{actn}_{1,4}\,[\mathrm{Bmal1}](t-\tau_1)}{\mathrm{act}_{1,4}}+1}{\frac{[\mathrm{Bmal1}](t-\tau_1)}{\mathrm{act}_{1,4}}+1}\right)^2 \left(\frac{1}{\frac{[\mathrm{Per2}](t-\tau_3)}{\mathrm{inh}_{3,4}}+1}\right)^2 \left(\frac{\frac{\mathrm{actn}_{5,4}\,[\mathrm{Dbp}](t-\tau_5)}{\mathrm{act}_{5,4}}+1}{\frac{[\mathrm{Dbp}](t-\tau_5)}{\mathrm{act}_{5,4}}+1}\right)\left(\frac{1}{\frac{[\mathrm{Cry1}](t-\tau_4)}{\mathrm{inh}_{4,4}}+1}\right)^2 - \mathrm{d4}\,[Cry1]\,(t)$$

$$\frac{d\,[Dbp]}{dt} = \left(\frac{\frac{\mathrm{actn}_{1,5}\,[\mathrm{Bmal1}](t-\tau_1)}{\mathrm{act}_{1,5}}+1}{\frac{[\mathrm{Bmal1}](t-\tau_1)}{\mathrm{act}_{1,5}}+1}\right)^3 \left(\frac{1}{\frac{[\mathrm{Per2}](t-\tau_3)}{\mathrm{inh}_{3,5}}+1}\right)^3 \left(\frac{1}{\frac{[\mathrm{Cry1}](t-\tau_4)}{\mathrm{inh}_{4,5}}+1}\right)^3 - \mathrm{d5}\,[Dbp]\,(t)$$

The following Table S1-1 lists how parameter names used in this paper (top) correspond to the names used in the original publication (bottom).

delay parameters	τ_1<br>τ_{Bmal1}	τ_2<br>$\tau_{Rev-erb\alpha}$	τ_3<br>τ_{Per2}	τ_4<br>τ_{Cry1}	τ_5<br>τ_{Dbp}
degradation rates	d_1<br>d_{Bmal1}	d_2<br>$d_{Rev-erb\alpha}$	d_3<br>d_{Per2}	d_4<br>d_{Cry1}	d_5<br>d_{Dbp}
kinetic parameters	$inh_{2,1}$<br>$ar1$	$inh_{2,4}$<br>$ar4$	$inh_{3,2}$<br>$cr2$	$inh_{3,3}$<br>$cr3$	$inh_{3,4}$<br>$cr4$
	$inh_{3,5}$<br>$cr5$	$inh_{4,2}$<br>$gr2$	$inh_{4,3}$<br>$gr3$	$inh_{4,4}$<br>$gr4$	$inh_{4,5}$<br>$gr5$
	$actn_{1,2}$<br>b_{RevErb}	$actn_{1,3}$<br>b_{Per2}	$actn_{1,4}$<br>b_{Cry1}	$actn_{1,5}$<br>b_{Dbp}	$actn_{5,2}$<br>f_{RevErb}
	$actn_{5,3}$<br>f_{Per2}	$actn_{5,4}$<br>f_{Cry1}	$act_{1,2}$<br>ba_2	$act_{1,3}$<br>ba_3	$act_{1,4}$<br>ba_4
	$act_{1,5}$<br>ba_5	$act_{5,2}$<br>fa_2	$act_{5,3}$<br>fa_3	$act_{5,4}$<br>fa_4	

Table S1-1: Corresponding parameter names in (Korenčič et al., 2014).

References

Bintu, L., Buchler, N. E., Garcia, H. G., Gerland, U., Hwa, T., Kondev, J., and Phillips, R. (2005). Transcriptional regulation by the numbers: models. *Curr Opin Genet Dev*, 15(2):116–124.

Höfer, T., Nathansen, H., Löhning, M., Radbruch, A., and Heinrich, R. (2002). GATA-3 transcriptional imprinting in Th2 lymphocytes: a mathematical model. *Proceedings of the National Academy of Sciences*, 99(14):9364–9368.

Korenčič, A., Bordyugov, G., Košir, R., Rozman, D., Goličnik, M., and Herzel, H. (2012). The interplay of cis-regulatory elements rules circadian rhythms in mouse liver. *PLoS One*, 7(11):e46835.

Korenčič, A., Košir, R., Bordyugov, G., Lehmann, R., Rozman, D., and Herzel, H. (2014). Timing of circadian genes in mammalian tissues. *Sci Rep*, 4:5782.

Ukai-Tadenuma, M., Yamada, R. G., Xu, H., Ripperger, J. A., Liu, A. C., and Ueda, H. R. (2011). Delay in feedback repression by cryptochrome 1 is required for circadian clock function. *Cell*, 144(2):268–281.
