# Supplementary material for: Feedback Loops of the Mammalian Circadian Clock Constitute Repressilator
Source: PLoS Comput Biol. 2016 Dec 12;12(12):e1005266. doi: 10.1371/journal.pcbi.1005266 (PMC5189953; doi:10.1371/journal.pcbi.1005266)
Supplement: S2 Appendix — Details on period jumps observed for specific parameter variations. (PDF) [file pcbi.1005266.s002.pdf]

S2 Bifurcations in the core clock model

Systematic parameter variation generally confirmed the robustness of the model. Bifurcation points and disruptions of normal circadian oscillations occurred in a reasonable distance to the default parameter configuration (Korenčič et al. (2014)). For some parameters, however, interesting phenomena such as period jumps were observed, which are described in more detail below.

Bifurcation diagrams allow to characterize these phenomena induced by parameter variations. Furthermore, in combination with targeted clamping (as described in S3 Appendix), loops can be identified that play an essential role before and after the period jumps.

Bifurcation diagrams were generated using adiabatic parameter changes[1] in both directions with 600 points each. This procedure allows to detect hysteresis, i.e. coexisting attractors close to bifurcations.

Per2 **delay**

For an increasing delay of *Per2*, the period is first rising as expected from the longer time needed to pass through the repressilator loop. Increasing the delay further, however, leads to a sudden jump of the period to a smaller value from which it is rising again to the original value and above (Figure **S2-1**). Notably, the phase relations of the genes are largely disturbed close to the jump, but are restored when the period reaches its original value.

Clamping before and after the jump reveals, that the repressilator plays an essential role, both before and after the jump. In particular, clamping any of the edges belonging to the repressilator disrupts oscillations completely. Interestingly, the strongly delayed (*Per2* ⊣ *Rev-erb-α*)-process influences a subsequent period of oscillations after the period jump, leading to a shortened period.

The bifurcation diagram (Figure **S2-1**) suggests that there is birhythmicity with coexisting limit cycles just when the jump occurs: Hysteresis is observed using the adiabatic parameter changes in both directions. The two limit cycles are shown in Figure **S2-2**.

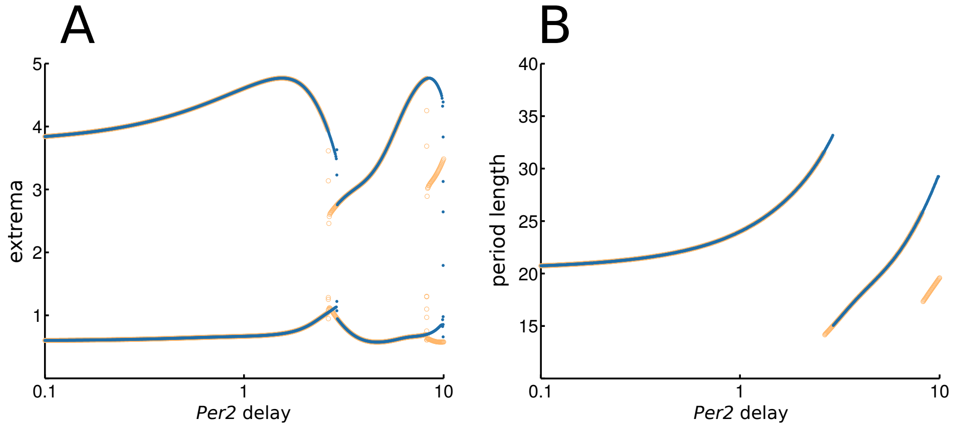

Figure S2-1: Maxima and minima (A) and periods (B) for *Per2* delay variation. Blue symbols refer to increasing parameters, whereas orange symbols refer to the reverse parameter variation.

[1] i.e. taking the end point of one parameter simulation as initial condition for the next parameter simulation

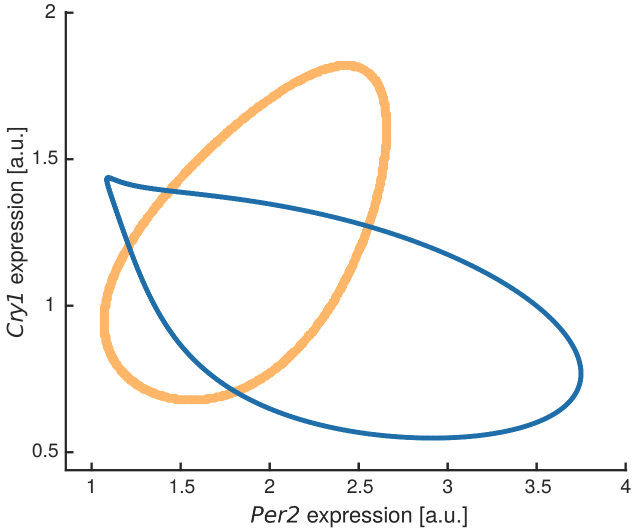

Figure S2-2: Phase portrait showing two coexisting limit cycles in the transition region before and after the period jump that occurs when the *Per2* delay is increased. Adiabatic simulation for both increasing (blue) and decreasing (orange) parameter values allows to find these different limit cycles for the same parameter value.

Cry1 degradation

When the *Cry1* degradation rate is increased, a period jump to a short period of about 10 hours happens (Figure **S2-3**). Clamping reveals, that the repressilator is not relevant anymore for oscillations that occur after the period jump. These low-period rhythms are mainly generated by *Cry1* self-inhibition.

At the transition between the loops is a region where complex rhythms can be detected. Time-series, next-maximum-plot and phase portrait confirm the existence of a superposition of two oscillations, a torus (Figure **S2-4**).

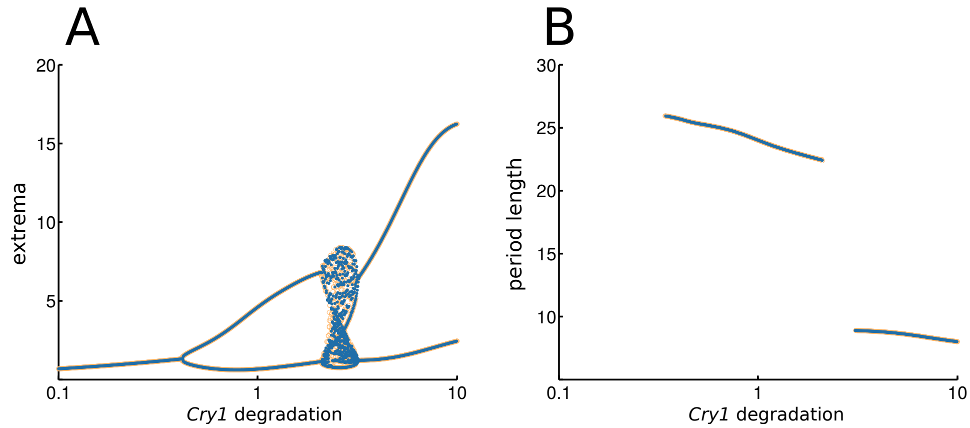

Figure S2-3: Maxima and minima (A) and periods (B) for *Cry1* degradation rate variation. Blue symbols refer to increasing parameters, whereas orange symbols refer to the reverse parameter variation.

Cry1 ⊣ *Per2*

When the *Per2* inhibition parameter is increased, a period jump to a short period of about 15 hours occurs (Figure **S2-5**). Using clamping we identified a *Per2*—*Dbp*—*Cry1* negative double-loop to be responsible for

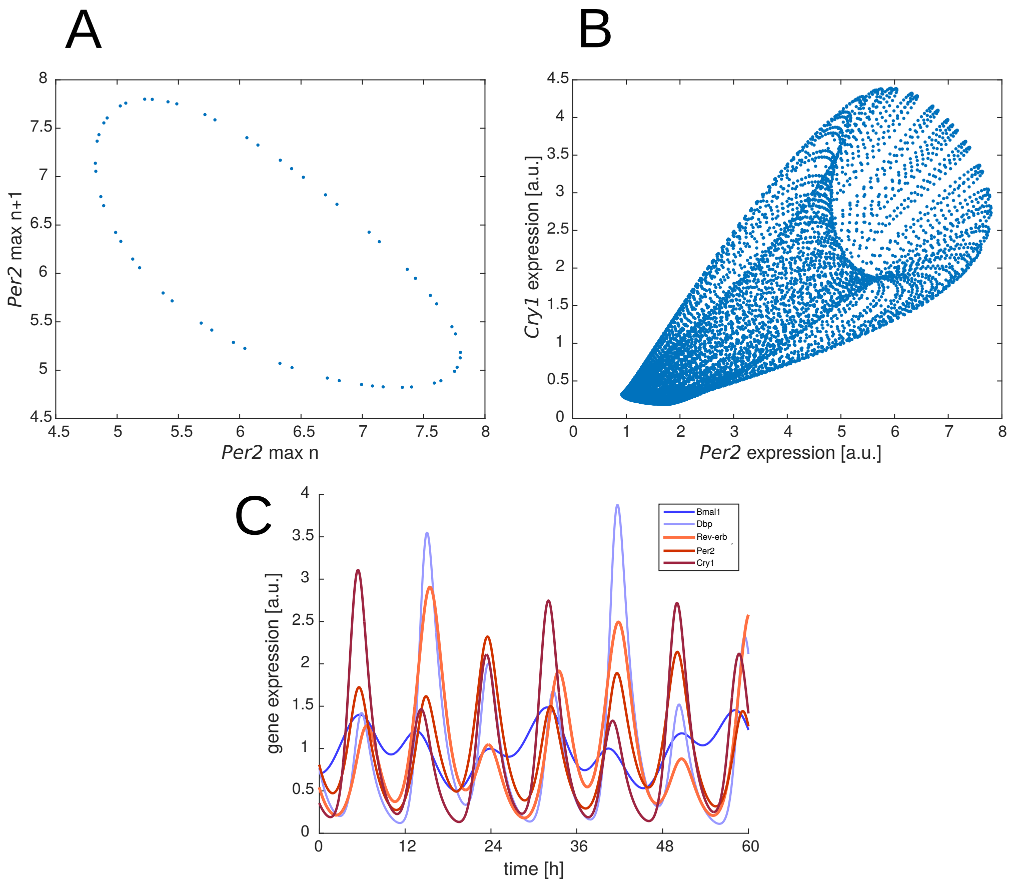

Figure S2-4: Next-maximum-plot (A), phase-portrait (B) and timeseries (C) for a *Cry1* degradation rate of 0.612. The figures indicate the superposition of two periods, i.e. a two-dimensional torus (see Berge et al. (1984) for an introduction to nonlinear phenomena).

the generation of oscillations. Either of the loops, *Per2*—*Dbp* or *Dbp*—*Cry1*, alone is not able to generate rhythms. Only in combination they create a feedback that is strong enough to trigger oscillations. This double-negative feedback loop design has been discussed previously as a robust oscillator by Kim and Forger (2012). As discussed in S4 Appendix, the period length is governed by the delay within the relevant loop. Thus it is not surprising that the short *Cry1* self-inhibition leads to a period of 10 hours, whereas the double-loop leads to a period of 15 hours.

There is also a region without oscillation in between the two rhythmic regimes. In this case, the bifurcation diagram indicates the existence of two supercritical Hopf-bifurcations with a steady state in between.

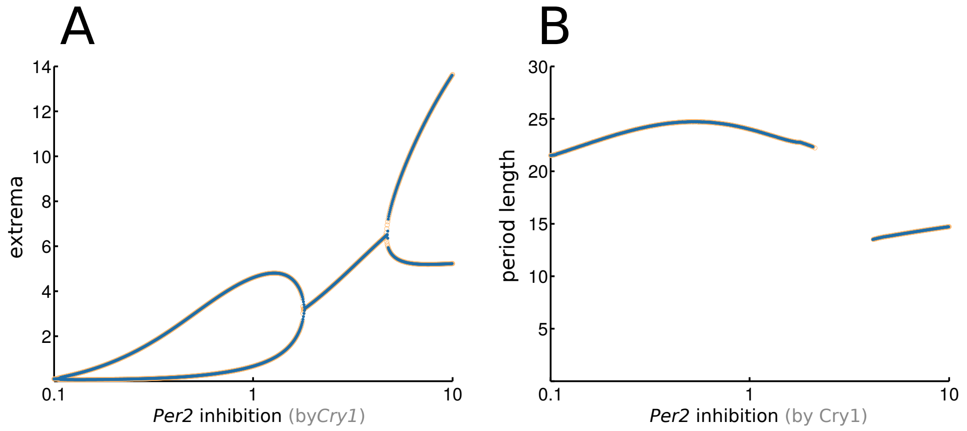

Figure S2-5: Maxima and minima (A) and periods (B) for *Per2* inhibition variation.

Bmal1 → *Rev-erb-α*

For variation of the activation strength of *Bmal1* on *Rev-erb-α*, we observe a supercritical Hopf-bifurcation (Figure **S2-6**).

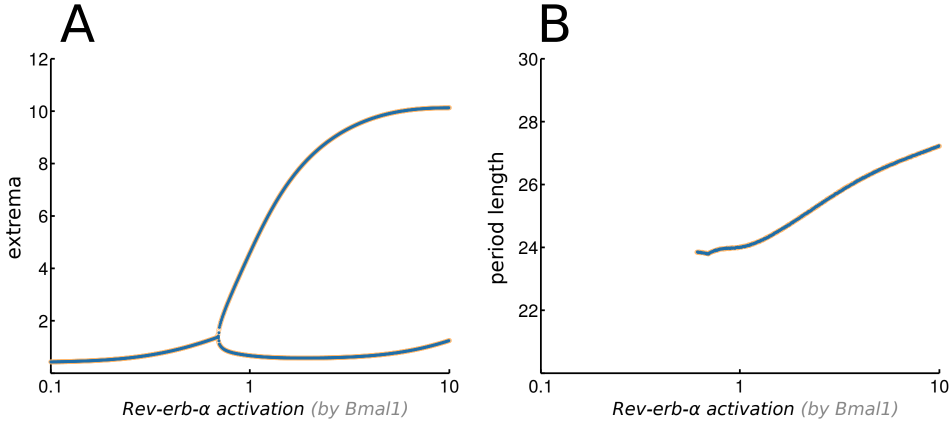

Figure S2-6: Maxima and minima (A) and periods (B) for *Rev-erb-α* activation variation.

References

Berge, P., Pomeau, Y., and Vidal, C. (1984). *Order within chaos.* Hermann, Paris.

Kim, J. K. and Forger, D. B. (2012). A mechanism for robust circadian timekeeping via stoichiometric balance. *Molecular Systems Biology*, 8(1):630.

Korenčič, A., Košir, R., Bordyugov, G., Lehmann, R., Rozman, D., and Herzel, H. (2014). Timing of circadian genes in mammalian tissues. *Sci Rep*, 4:5782.
