# Supplementary material for: Feedback Loops of the Mammalian Circadian Clock Constitute Repressilator
Source: PLoS Comput Biol. 2016 Dec 12;12(12):e1005266. doi: 10.1371/journal.pcbi.1005266 (PMC5189953; doi:10.1371/journal.pcbi.1005266)
Supplement: S3 Appendix — Details on the clamping procedure extensively used in this work. (PDF) [file pcbi.1005266.s003.pdf]

S3 Combinatorial analysis of sub-models

Motivation

To analyze model function more deeply, we devise a combinatorial clamping strategy. Exclusion of some parts of the model allows us to examine the independent function of their respective complement. Clamping parts of the model—instead of removal—ensures that the conditions to which the model was fitted remain largely constant. For example, a basal activating effect will not be removed completely, but just set constant. However, clamped genes will not be able to transmit variations in gene expression.

Thus, in the context of circadian rhythms, they can also not constitute a part of an active oscillation generating mechanism. We clamp both genes and processes, corresponding to nodes and edges of the network graph, respectively.

Here, we explain in detail how clamping in the analyzed circadian core clock model was done. We will first describe the clamping of genes and then the clamping of processes. Finally, insights on how oscillations are generated in the model will be presented.

Clamping genes

Examination of sub-networks of the full consensus core clock model (Korenčič et al., 2014) was done by systematically clamping all possible combinations of genes. An overview of all $2^5 = 32$ combinations of the 5 genes is given in Figure **S3-1**, with oscillatory sub-models marked in yellow.

On the level of differential equations, clamping is implemented by saving the mean value of all genes first. The mean value is taken after a sufficiently long transient of the unmodified model with default parametrization. Then, clamping a gene to its mean value means to replace all occurrences of that gene in the differential equations of other genes by its constant mean value.

Alternatively, averages of the regulation terms could be considered. We compared the two averages and found that the differences are small in most cases. In a few situations the differences are larger due to large amplitudes of *Rev-erb-α*, but we confirmed that our main results do not depend on the averaging method.

Example S3-1 Clamping *Bmal1* would mean to replace the parts marked blue in the differential equation of *Cry1* by the mean value of *Bmal1*, and analogously in all other equations.

$$\frac{d[Cry1]}{dt} = \left(\frac{1}{\frac{[RevErb_\alpha]_\tau}{\mathrm{ar4}}+1}\right)^2 \left(\frac{\frac{\mathrm{b\_Cry1}\,[Bmal1]_\tau}{\mathrm{ba4}}+1}{\frac{[Bmal1]_\tau}{\mathrm{ba4}}+1}\right)^2 \left(\frac{1}{\frac{[Per2]_\tau}{\mathrm{cr4}}+1}\right)^2 \left(\frac{\frac{\mathrm{f\_Cry1}\,[Dbp]_\tau}{\mathrm{fa4}}+1}{\frac{[Dbp]_\tau}{\mathrm{fa4}}+1}\right) \left(\frac{1}{\frac{[Cry1]_\tau}{\mathrm{gr4}}+1}\right)^2 - \mathrm{d4}\,[Cry1]$$

Using this method of clamping, all combinatorial subsets of genes were examined for their ability to generate oscillations (Figure **S3-1**). It is still quite likely that oscillations vanish in a subset of genes to which the model parameters were not optimized. In order to examine the rhythm generating potential of subsets more deeply, we explore parameter variations around the default value. To this end, every sub-model given by a specific subset of genes was subjected to parameter variations from five-fold decrease to five-fold increase of the default value. The configurations with sustained oscillations were counted, and selected bar plots depicting their frequencies are shown in Figure 3. The complementary bar plot for gene groups of size 3 is shown in Figure **S3-2**. If at least one oscillating solution was found in this range, the respective subset of genes is marked yellow in Figure **S3-1**. A expected, less oscillating solutions are found for smaller subsets.

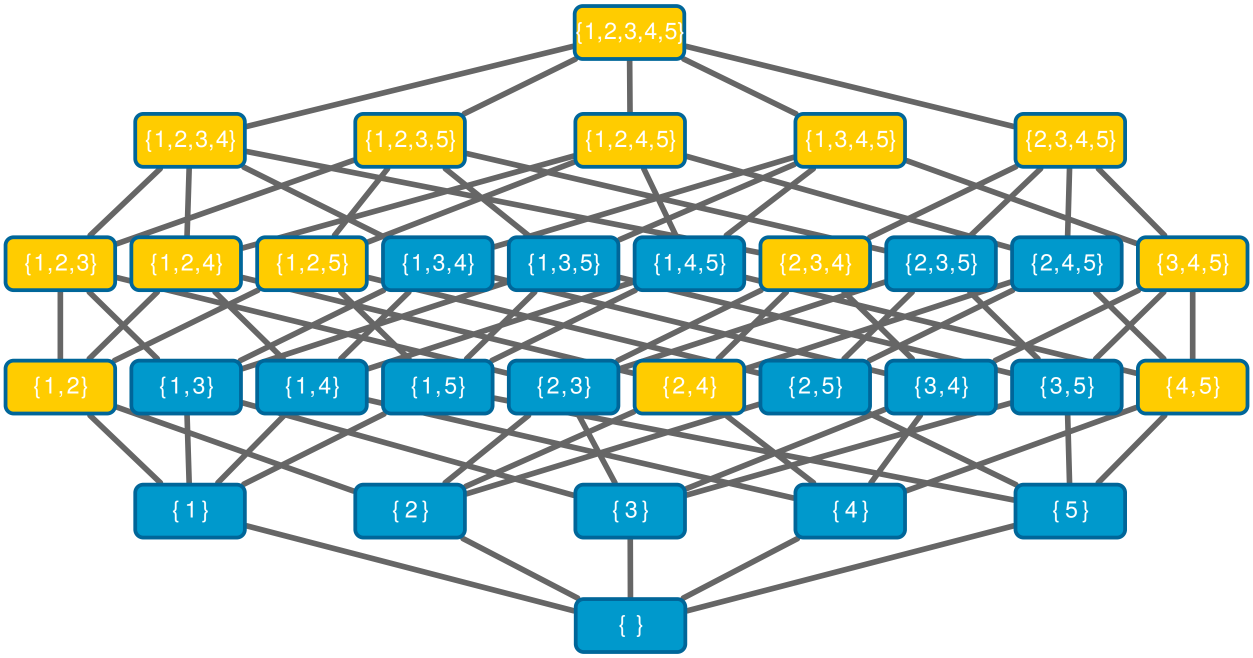

Figure S3-1: All $2^5 = 32$ subsets of the 5 modeled genes. Numbers correspond to: 1–$Bmal1$, 2–$Rev-erb-\alpha$, 3–$Per2$, 4–$Cry1$, 5–*Dbp*. If at least one oscillating solution was found for a subset, it is marked yellow.

Individual examination. For sub-models found to be capable of generating oscillations relevant parameters, their values and the resulting periods were examined further. Particularly notable—as stated in the main text—are the small subsets comprising {*Rev-erb-α*, *Bmal1*} and {*Per2*, *Cry1*, *Rev-erb-α*}. The former is able to generate 24h rhythms after just doubling the parameter corresponding to *Rev-erb-α* activation by *Bmal1*, while the latter is able to generate oscillations without any modification of the default parameter values at all.

Clamping processes

Analogously to clamping genes, processes were clamped, allowing us to examine the importance of particular edges in the network graph of the model. There are $2^{17} = 131072$ possible combinations of the 17 edges that can be examined. We term a vector $c \in \{0,1\}^{17}$ that assigns a state ON (1) or OFF (0) to each edge ON/OFF-configuration. OFF means being clamped, while ON means not being clamped. As for clamping genes, we take the mean value of the unmodified model with default parametrization after a sufficiently long transient first.

Clamping a process to its mean value then means to replace the expression value of the regulating gene in the differential equation of the target gene by its mean value. E.g. if v_1 and v_2 are genes and the edge (v_1, v_2) describes a process of v_1 regulating v_2, then clamping (v_1, v_2) means to fix v_1 in the differential equation of v_2 to the previously measured mean value of v_1. In fact, clamping all processes $P_i = \{(v_i, v_j) \mid j \in 1, \ldots, 5; (v_i, v_j) \in E\}$ from source gene v_i to all target genes v_j corresponds to clamping gene v_i as described in the previous section. However, the analysis is not redundant with the previous section, as we do not examine parameter changes of all 2^{17} ON/OFF-configurations here.

Again we systematically test all combinations of processes being clamped (i.e. all ON/OFF-configurations) to examine their ability of generating oscillations. In this way individual edges can be excluded from being part of an essential rhythm generating mechanism. The resolution is high enough to identify network motifs as minimal rhythm generating core units. From theory, it is known that these motifs must be negative feedback loops (Thomas et al., 1995).

We assign a score to each edge in the network graph by calculating the frequency with which it occurs in

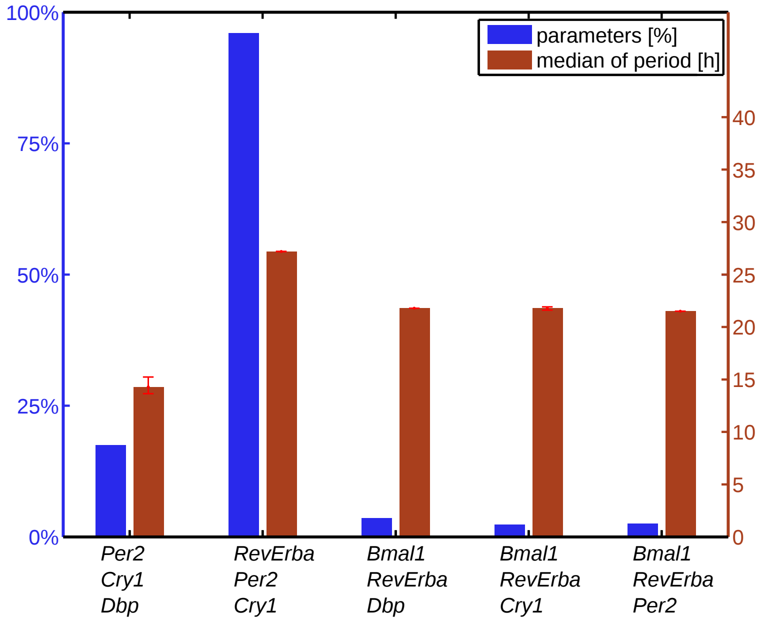

Figure S3-2: Gene subsets of 3 genes for which oscillations were found after varying parameters from one fifth to five-fold the default values. Blue bars indicate the percentage of parameter variations for which oscillations were found. The repressilator motif in the second sub-network (*Rev-erb-*α, *Per2*, *Cry1*) is oscillating without parameter changes, and only a few changes can stop oscillations (value below 100%), while the median period after variation is about 27h. In the sub-networks to the right of the repressilator, a particular change activated the *Bmal1*–*Rev-erb-*α loop and triggered oscillations with slightly less than 24h median period. In the sub-network to the left of the repressilator, small period oscillations are caused by activation of different *Per2*–*Dbp*–*Cry1* negative feedback loops.

the set of oscillating ON/OFF-configurations. There are 14125 oscillating ON/OFF-configurations in total. This calculation is illustrated in Example **S3-2**.

Example S3-2 M is the matrix of all oscillating ON/OFF-configurations. Each row corresponds to one ON/OFF-configuration describing for each of the 17 processes whether it is clamped (0) or not (1). The frequency of each process of occurring in an oscillating ON/OFF-configuration can be obtained by summing up the columns of M and dividing the resulting vector elementwise by the total number of oscillating configurations.

$$M = \begin{pmatrix} 0 & 0 & 1 & 0 & 0 & 1 & 1 & 0 & 0 & 0 & 0 & 1 & 0 & 0 & 0 & 0 & 0 \\ 0 & 1 & 0 & 0 & 0 & 1 & 1 & 1 & 1 & 1 & 0 & 1 & 0 & 1 & 0 & 0 & 0 \\ 0 & 1 & 1 & 0 & 0 & 1 & 1 & 1 & 0 & 1 & 1 & 1 & 0 & 0 & 1 & 0 & 0 \\ 1 & 0 & 1 & 0 & 0 & 1 & 1 & 1 & 1 & 0 & 0 & 1 & 1 & 1 & 1 & 0 & 0 \\ 1 & 1 & 1 & 0 & 1 & 1 & 1 & 1 & 1 & 1 & 0 & 1 & 1 & 0 & 0 & 1 & 0 \\ 0 & 1 & 0 & 1 & 1 & 1 & 1 & 0 & 0 & 1 & 1 & 1 & 1 & 1 & 0 & 1 & 0 \\ 1 & 0 & 0 & 0 & 0 & 1 & 1 & 0 & 1 & 0 & 0 & 1 & 0 & 1 & 1 & 1 & 0 \\ 0 & 0 & 1 & 1 & 0 & 1 & 1 & 0 & 0 & 0 & 1 & 1 & 0 & 0 & 0 & 0 & 1 \\ 1 & 1 & 0 & 1 & 1 & 1 & 1 & 0 & 1 & 1 & 1 & 1 & 0 & 1 & 0 & 0 & 1 \\ 0 & 0 & 0 & 1 & 1 & 1 & 1 & 1 & 1 & 0 & 0 & 1 & 1 & 0 & 1 & 0 & 1 \\ \vdots & \vdots & \vdots & \vdots & \vdots & \vdots & \vdots & \vdots & \vdots & \vdots & \vdots & \vdots & \vdots & \vdots & \vdots & \vdots & \vdots \\ 0 & 1 & 0 & 1 & 0 & 1 & 1 & 0 & 0 & 0 & 1 & 1 & 1 & 1 & 1 & 0 & 1 \\ 0 & 0 & 0 & 0 & 0 & 1 & 1 & 1 & 0 & 1 & 0 & 1 & 1 & 0 & 0 & 1 & 1 \\ 1 & 0 & 1 & 1 & 0 & 1 & 1 & 1 & 0 & 0 & 1 & 1 & 1 & 1 & 0 & 1 & 1 \\ 0 & 1 & 0 & 0 & 0 & 1 & 1 & 1 & 1 & 1 & 0 & 1 & 1 & 0 & 1 & 1 & 1 \end{pmatrix}$$

Visualization of the scores is presented in Figure 5 of the main text. It becomes apparent that one negative feedback loop, a repressilator, is responsible for generating most of the found oscillations. This repressilator appears in 97% of the oscillating ON/OFF-configurations. Indeed, all other processes can be clamped and the repressilator still generates rhythms under the default parametrization.

Conclusions

The analyses described in the previous two sections, together with further exploration of the parameter space, led us to the following description how rhythms are generated in the model:

Repressilator generates oscillations. The repressilator motif generates rhythms, even after clamping all other edges that are not part of it and clamping of a single repressilator edge stops oscillations. Clearly, the repressilator appears as the driving force of rhythms in the model.

However, the generated period (36h) is comparably long. This is not surprising, since the kinetic parameter values were fitted for the complete model to generate 24h rhythms. Reducing the model drastically counteracts this optimization, leading to a long period.

Other processes adjust the period. Indeed, inclusion of additional edges to the isolated repressilator motif reduces the generated period step by step. This is achieved by reducing the implicit delay of the loop. For example, rhythmic *Bmal1* and *Dbp* genes help to activate the corresponding repressilator genes at the right time and, thereby, reduce the time needed to continue the cycle of oscillations. Self-inhibition of *Per2* and *Cry1*, respectively, reduces the time needed for inactivation of repressilator genes, and further reduces the implicit delay. Thus, many components may not be essential for the generation of rhythms, but yet contribute to the function of the model by tuning the period.

Interestingly, the reduction of period by inclusion of additional regulations happens subadditively[1]. Adding *Bmal1*, *Dbp*, or both self-inhibitions alone, reduces the period from 36h to about 30h. Adding a second of these elements reduces it to less than 27h. Adding all edges finally results in the complete model generating 24h rhythms. Reversely, this means that single modifications of the complete model result in

[1]adding several components simultaneously has a smaller effect than the sum of effects of each component added alone

minor changes of the period, which can be interpreted as a sign of robustness. Redundancy in the regulation of period, pushing it towards 24h, accounts for the observed subadditive effect.

The contribution of non-essential but fine-tuning redundant processes seems to be a plausible general design principle for adding robustness to a system.

Consistent with the described mechanism is the observation, that perturbations of circadian genes via RNAi silencing of circadian regulators often lead to period lengthening (Maier et al., 2009).

REV-ERB–BMAL1 loop is a potential circadian oscillator. Further, the *Rev-erb-α–Bmal1* is also able to generate 24h rhythms in the model alone, if the activation strength of *Bmal1* → *Rev-erb-α* is doubled, which constitutes only a minor adjustment. Then, the period is quite robust to further variations of kinetic parameters (compare Figure 2C of the main text).

PER and CRY single loops were not found to oscillate with a circadian period. The two single-gene self-inhibitions of *Per2* and *Cry1* in the model were not found to generate a circadian period, but smaller periods of about 10h were found, which is due to the relatively small delays. However, the repressilator, which is able to generate large period oscillations, is coherent with these self-inhibitions.

References

Korenčič, A., Košir, R., Bordyugov, G., Lehmann, R., Rozman, D., and Herzel, H. (2014). Timing of circadian genes in mammalian tissues. *Sci Rep*, 4:5782.

Maier, B., Wendt, S., Vanselow, J. T., Wallach, T., Reischl, S., Oehmke, S., Schlosser, A., and Kramer, A. (2009). A large-scale functional rnai screen reveals a role for ck2 in the mammalian circadian clock. *Genes & Development*, 23:708–718.

Thomas, R., Thieffry, D., and Kaufman, M. (1995). Dynamical behaviour of biological regulatory networks–i. biological role of feedback loops and practical use of the concept of the loop-characteristic state. *Bull Math Biol*, 57:247–276.
