# Supplementary material for: Feedback Loops of the Mammalian Circadian Clock Constitute Repressilator
Source: PLoS Comput Biol. 2016 Dec 12;12(12):e1005266. doi: 10.1371/journal.pcbi.1005266 (PMC5189953; doi:10.1371/journal.pcbi.1005266)
Supplement: S4 Appendix — Overview and analysis of the relation of loops in the core clock network. (PDF) [file pcbi.1005266.s004.pdf]

S4 Topological structure:
Feedback and feedforward loops in the core clock network

Topological structure of the network

High connectivity and large amount of negative feedback loops. The network graph studied here was assembled from biological evidence of transcription factor binding sites (E-boxes, D-boxes and RREs) of core clock genes (Ukai and Ueda, 2010; Korenčič et al., 2014). It possesses a high density of regulations, as 17 out of 25 possible edges are implemented.

Moreover, it also contains a large number of feedback loops: 2 of the 5 genes feed back to their own transcription and 5 out of 10 gene-pairs form a loop. Furthermore, 6 of 10 gene-triplets and 4 of 15 quadruples contain loops. A complete overview of all negative and positive feedback loops is given in Tables **S4-1** and **S4-2**, respectively.

This large amount of loops points towards multiple possible oscillators and a potentially complex dynamical behaviour of the model. Most studies focus on two of these negative feedback loops: The self-inhibitory PER/CRY loop and a loop comprising REV-ERB and BMAL1.

Analyzing the organization of loops. Due to the small size and high density of the network, a classical analysis of "network motifs" as introduced by (Milo et al., 2002) seems inappropriate. First, the large amount of present edges leads to complex motifs, most of which are unique in the network. The function of these complex motifs is difficult to interpret. Second, the overall amount of countable motif occurrences is low in the small network. Thus, no statistics can be reasonably applied on the set of motifs, which would be necessary for overrepresentation analysis.

We therefore develop a different approach to reveal the structure of the network: Focusing on feedback and feedforward loops as basic elements, we list all of them and analyze their interconnection through common edges. Feedback loops represent means by which information recurs to the same node, while feedforward loops represent means by which information is passed on to another node. Hence, those two instances and their combinations describe how signals are processed in the network.

If a given negative feedback loop is extended by adding a link via another node, this link bypasses a link of the original loop. Then, the two coexisting links form a feedforward loop, which can be coherent (same sign) or incoherent (different sign). The original feedback loop and its extended version are thus connected via a feedforward loop and related to each other. Coherent feedforward loops induce redundancy. If they appear within a negative feedback loop they can increase the robustness of rhythm generation. Different feedback loops related in this way can support rather than obstruct each other. This type of redundancy might also constitute a design principle of robust oscillators.

As Table **S4-2** shows, a number of positive feedback loops is found in the network as well. Interestingly, also positive feedback loops can contribute to the generation of oscillations by reducing the minimal Hill-coefficients of negative feedback loops to generate oscillations (Ananthasubramaniam and Herzel, 2014). Thus very large Hill coefficients, often regarded as unphysiological, can be circumvented. But positive feedback loops can also be obstructive for rhythm generation by counteracting negative feedback loops, depending on the timing of involved processes. Thus, the study of timing and strength of processes will be complementary to topological analyses to fully understand the mechanisms determining the system's behaviour.

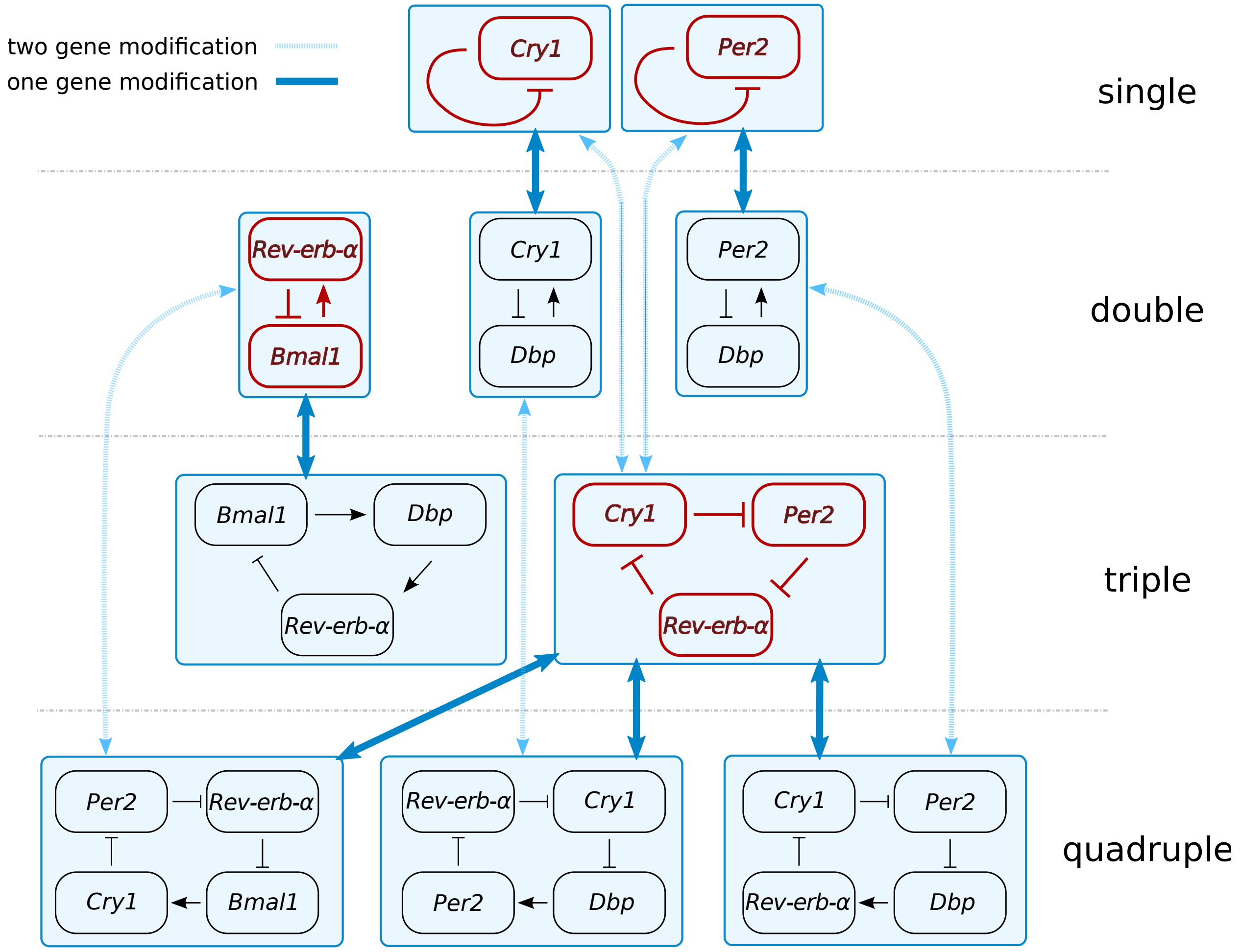

Figure S4-1: Relation of negative feedback loops of size 1 to 4 in the core clock network. Prominent loops discussed in this paper are marked in red. One size 4 loop is not depicted, which is weakly connected to the rest of the network (compare Table **S4-3**).

Negative feedback loops are closely related. It turns out that larger loops often contain smaller loops as a sub-part and just add one or two elements. For example, the *Cry1* and *Per2* self-inhibition can be extended by including *Dbp*. A map of the interrelations of negative feedback loops can be constructed and is shown in Figure **S4-1**.

As negative feedback loops are a necessary condition for the generation of oscillations (Thomas et al., 1995), we focus here on understanding their organization in the network. Negative feedback loops emerge from the interplay of at least one negative regulator (*Per2*, *Cry1*, *Rev-erb-α*) and positive regulators (*Bmal1*, *Dbp*). Since inclusion of a positive regulation does not change the sign of a feedback loop, negative loops can be extended easily by including additional positive links. To extend a loop using negative regulations, an even number of links has to be added, resulting in an overall positive regulation.

All negative feedback loops we found can be transformed into others by adding or removing a single regulator. An overview of which negative feedback loops can be derived from each other by addition or deletion of additional regulators is shown in Figure **S4-1** and a complete list is given in Table **S4-3**.

The observed high interrelation questions the distinction of individual loops. All found negative feedback loops appear rather as a coherent multi-loop-construct on the topological level. To describe their coherence more quantitatively, timescales of the involved processes have to be taken into account.

Central location of the repressilator. The repressilator motif discussed in this paper appears in the middle of this hierarchy and connects a larger number of motifs (see Figure **S4-1**). It is related to coherent loops of smaller size, such as the PER/CRY self inhibitory loops, as well as to a number of larger feedback loops. Figure **S4-2** shows how additional positive regulators can provide coherent feedforward links to edges of the repressilator. In this way Figure **S4-2** gives a condensed representation of larger coherent loops.

So far we discussed only the topology of feedback loops. To evaluate generation of 24h oscillations, the delay has to be considered. Mathematical theory predicts that the delay should be in the range of 6h to 12h for circadian rhythms (MacDonald et al., 2008; Bordyugov et al., 2013). The delays of the *Per2* and *Cry1* self-inhibition are only 3.82h and 3.13h in the model and are thus not large enough to generate 24h oscillations.

The medium size of the repressilator, however, allows delays of about 3-4h for each link. These add up to a sufficiently large overall delay, resulting in oscillations with a 24h period (see Table **S4-1**). This is similarly fulfilled by the REV-ERB–BMAL1 loop, which was also found to play an important role in the model.

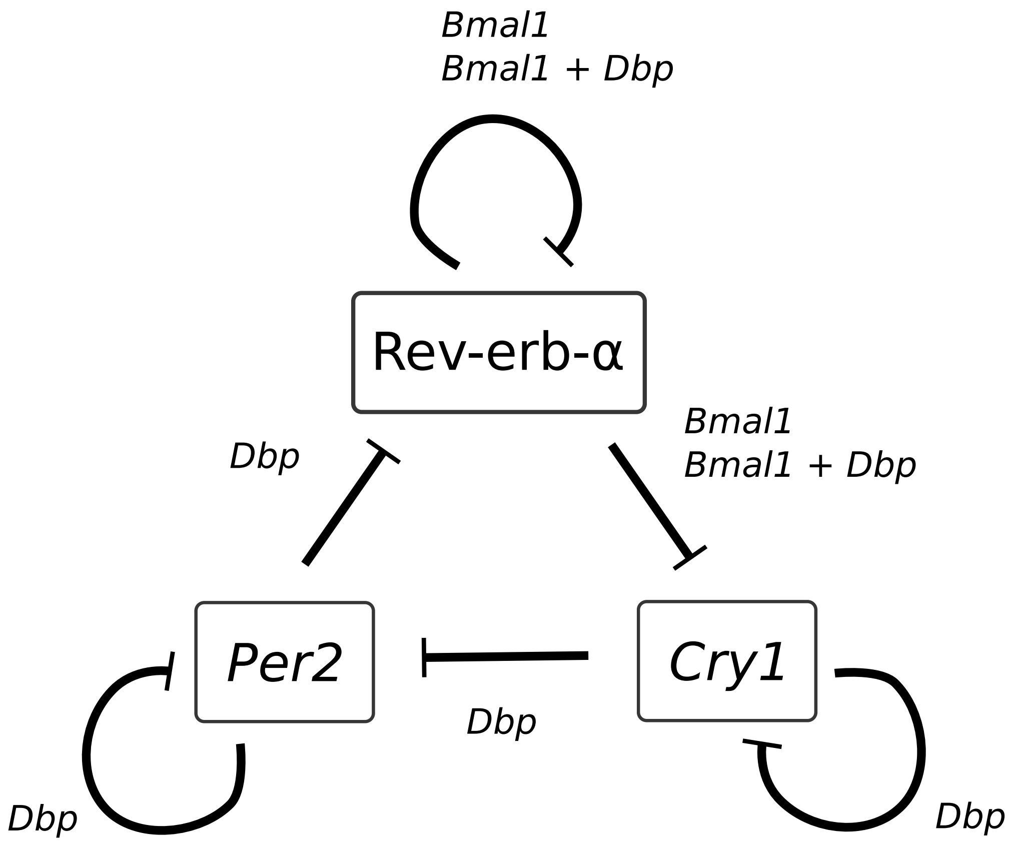

Figure S4-2: The repressilator as a representative motif, with coherent alternative links via *Dbp* and *Bmal1* listed at each edge. A direct regulation exists for all links, except for *Rev-erb-α* auto-inhibition. Note, that each of the three single gene loops is also coherent with the repressilator. It emerges that *Dbp* plays a particularly important role in providing additional links. Since its delay in the model is small with about 2h, these links are also more likely to be temporally coherent.

Summary. The comprehensive network analysis reveals multiple positive and negative feedback loops. It turns out that some of them including the repressilator exhibit the appropriate delay to generate 24h rhythms. Interestingly, small negative loops are often extended by coherent feedforward loops. This design principle increases the robustness and contributes to the fine-tuning of the period.

Table S4-1: **Table of negative feedback loops.** Prominent loops discussed in this paper are marked in red.

no.		cycle	delay
1	size 1	*Cry1* ⊣ *Cry1*	3.13
2		*Per2* ⊣ *Per2*	3.82
3	size 2	*Dbp* → *Cry1* ⊣ *Dbp*	5.21
4		*Dbp* → *Per2* ⊣ *Dbp*	5.90
5		*RevErba* ⊣ *Bmal1* → *RevErba*	6.55
6	size 3	*Cry1* ⊣ *Per2* ⊣ *RevErba* ⊣ *Cry1*	8.74
7		*Dbp* → *RevErba* ⊣ *Bmal1* → *Dbp*	8.63
8	size 4	*Dbp* → *Per2* ⊣ *RevErba* ⊣ *Cry1* ⊣ *Dbp*	10.82
9		*Dbp* → *RevErba* ⊣ *Cry1* ⊣ *Per2* ⊣ *Dbp*	10.82
10		*Cry1* ⊣ *RevErba* ⊣ *Bmal1* → *Per2* ⊣ *Cry1*	13.50
11		*Cry1* ⊣ *Per2* ⊣ *RevErba* ⊣ *Bmal1* → *Cry1*	13.50
12	size 5	*Dbp* → *RevErba* ⊣ *Bmal1* → *Per2* ⊣ *Cry1* ⊣ *Dbp*	15.58
13		*Dbp* → *Per2* ⊣ *RevErba* ⊣ *Bmal1* → *Cry1* ⊣ *Dbp*	15.58
14		*Dbp* → *RevErba* ⊣ *Bmal1* → *Cry1* ⊣ *Per2* ⊣ *Dbp*	15.58
15		*Dbp* → *Cry1* ⊣ *RevErba* ⊣ *Bmal1* → *Per2* ⊣ *Dbp*	15.58
16		*Dbp* → *Per2* ⊣ *Cry1* ⊣ *RevErba* ⊣ *Bmal1* → *Dbp*	15.58
17		*Dbp* → *Cry1* ⊣ *Per2* ⊣ *RevErba* ⊣ *Bmal1* → *Dbp*	15.58

Table S4-2: **Table of positive feedback loops.**

no.		cycle	delay
1	size 2	*Cry1* ⊣ *Per2* ⊣ *Cry1*	6.95
2		*Cry1* ⊣ *RevErba* ⊣ *Cry1*	4.92
3	size 3	*Dbp* → *Per2* ⊣ *Cry1* ⊣ *Dbp*	9.03
4		*Dbp* → *Cry1* ⊣ *Per2* ⊣ *Dbp*	9.03
5		*Dbp* → *RevErba* ⊣ *Cry1* ⊣ *Dbp*	7.00
6		*Cry1* ⊣ *RevErba* ⊣ *Bmal1* → *Cry1*	9.68
7		*Per2* ⊣ *RevErba* ⊣ *Bmal1* → *Per2*	10.37
8	size 4	*Dbp* → *RevErba* ⊣ *Bmal1* → *Cry1* ⊣ *Dbp*	11.76
9		*Dbp* → *Cry1* ⊣ *RevErba* ⊣ *Bmal1* → *Dbp*	11.76
10		*Dbp* → *RevErba* ⊣ *Bmal1* → *Per2* ⊣ *Dbp*	12.45
11		*Dbp* → *Per2* ⊣ *RevErba* ⊣ *Bmal1* → *Dbp*	12.45

Table S4-3: **Extensions of negative feedback loops.** (compare no. with Table **S4-1**). Extension means a longer loop with the same elements in the same order and one or two additional elements pasted in at any position.

no.	extensions
1	*Cry1* ⊣ *Per2* ⊣ *RevErba* ⊣ *Cry1*<br>*Dbp* → *Cry1* ⊣ *Dbp*
2	*Cry1* ⊣ *Per2* ⊣ *RevErba* ⊣ *Cry1*<br>*Dbp* → *Per2* ⊣ *Dbp*
3	*Dbp* → *RevErba* ⊣ *Cry1* ⊣ *Per2* ⊣ *Dbp*<br>*Dbp* → *Per2* ⊣ *RevErba* ⊣ *Cry1* ⊣ *Dbp*
4	*Dbp* → *RevErba* ⊣ *Cry1* ⊣ *Per2* ⊣ *Dbp*<br>*Dbp* → *Per2* ⊣ *RevErba* ⊣ *Cry1* ⊣ *Dbp*
5	*Cry1* ⊣ *Per2* ⊣ *RevErba* ⊣ *Bmal1* → *Cry1*<br>*Cry1* ⊣ *RevErba* ⊣ *Bmal1* → *Per2* ⊣ *Cry1*<br>*Dbp* → *RevErba* ⊣ *Bmal1* → *Dbp*
6	*Dbp* → *Cry1* ⊣ *Per2* ⊣ *RevErba* ⊣ *Bmal1* → *Dbp*<br>*Dbp* → *RevErba* ⊣ *Bmal1* → *Cry1* ⊣ *Per2* ⊣ *Dbp*<br>*Dbp* → *Per2* ⊣ *RevErba* ⊣ *Bmal1* → *Cry1* ⊣ *Dbp*<br>*Cry1* ⊣ *Per2* ⊣ *RevErba* ⊣ *Bmal1* → *Cry1*<br>*Dbp* → *RevErba* ⊣ *Cry1* ⊣ *Per2* ⊣ *Dbp*<br>*Dbp* → *Per2* ⊣ *RevErba* ⊣ *Cry1* ⊣ *Dbp*
7	*Dbp* → *Cry1* ⊣ *Per2* ⊣ *RevErba* ⊣ *Bmal1* → *Dbp*<br>*Dbp* → *Per2* ⊣ *Cry1* ⊣ *RevErba* ⊣ *Bmal1* → *Dbp*<br>*Dbp* → *Cry1* ⊣ *RevErba* ⊣ *Bmal1* → *Per2* ⊣ *Dbp*<br>*Dbp* → *RevErba* ⊣ *Bmal1* → *Cry1* ⊣ *Per2* ⊣ *Dbp*<br>*Dbp* → *Per2* ⊣ *RevErba* ⊣ *Bmal1* → *Cry1* ⊣ *Dbp*<br>*Dbp* → *RevErba* ⊣ *Bmal1* → *Per2* ⊣ *Cry1* ⊣ *Dbp*
8	*Dbp* → *Per2* ⊣ *RevErba* ⊣ *Bmal1* → *Cry1* ⊣ *Dbp*
9	*Dbp* → *RevErba* ⊣ *Bmal1* → *Cry1* ⊣ *Per2* ⊣ *Dbp*
10	*Dbp* → *Per2* ⊣ *Cry1* ⊣ *RevErba* ⊣ *Bmal1* → *Dbp*<br>*Dbp* → *Cry1* ⊣ *RevErba* ⊣ *Bmal1* → *Per2* ⊣ *Dbp*<br>*Dbp* → *RevErba* ⊣ *Bmal1* → *Per2* ⊣ *Cry1* ⊣ *Dbp*
11	*Dbp* → *Cry1* ⊣ *Per2* ⊣ *RevErba* ⊣ *Bmal1* → *Dbp*<br>*Dbp* → *RevErba* ⊣ *Bmal1* → *Cry1* ⊣ *Per2* ⊣ *Dbp*<br>*Dbp* → *Per2* ⊣ *RevErba* ⊣ *Bmal1* → *Cry1* ⊣ *Dbp*

References

Ananthasubramaniam, B. and Herzel, H. (2014). Positive feedback promotes oscillations in negative feedback loops. *PLoS One*, 9:e104761.

Bordyugov, G., Westermark, P. O., Korenčič, A., Bernard, S., and Herzel, H. (2013). Mathematical modeling in chronobiology. In *Circadian clocks*, pages 335–357. Springer.

Korenčič, A., Košir, R., Bordyugov, G., Lehmann, R., Rozman, D., and Herzel, H. (2014). Timing of circadian genes in mammalian tissues. *Sci Rep*, 4:5782.

MacDonald, N., Cannings, C., and Hoppensteadt, F. C. (2008). *Biological delay systems: linear stability theory*. Cambridge University Press.

Milo, R., Shen-Orr, S., Itzkovitz, S., Kashtan, N., Chklovskii, D., and Alon, U. (2002). Network motifs: simple building blocks of complex networks. *Science*, 298:824–827.

Thomas, R., Thieffry, D., and Kaufman, M. (1995). Dynamical behaviour of biological regulatory networks–i. biological role of feedback loops and practical use of the concept of the loop-characteristic state. *Bull Math Biol*, 57:247–276.

Ukai, H. and Ueda, H. R. (2010). Systems biology of mammalian circadian clocks. *Annu Rev Physiol*, 72:579–603.
