# Supplementary material for: Feedback Loops of the Mammalian Circadian Clock Constitute Repressilator
Source: PLoS Comput Biol. 2016 Dec 12;12(12):e1005266. doi: 10.1371/journal.pcbi.1005266 (PMC5189953; doi:10.1371/journal.pcbi.1005266)
Supplement: S5 Appendix — Details on the robustness comparison of repressilator and Goodwin oscillator described in the results section. (PDF) [file pcbi.1005266.s005.pdf]

S5 Robustness analysis:
Repressilator versus Goodwin oscillator

To support the claim that the repressilator (Figure **S5-1**) exhibits a higher robustness than the Goodwin oscillator (Figure **S5-2**), the following deterministic models are used to investigate the Hill coefficients. The equations are simplified by nondimensionalization and do not aim to represent a complex system close to their real counterparts. Instead, they are designed to be as simple as possible to facilitate comparison:

Figure S5-1: Repressilator

$$\frac{dx}{dt} = \frac{1}{1+z^m} - d_1 x$$

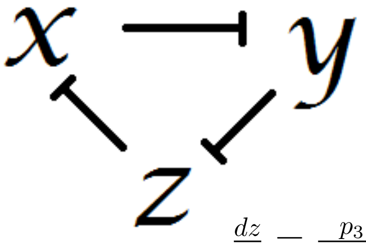

$$\frac{dz}{dt} = \frac{p_2}{1+x^m} - d_2 y$$

$$\frac{dz}{dt} = \frac{p_3}{1+y^m} - d_3 z$$

Figure S5-2: Goodwin oscillator

$$\frac{dx}{dt} = \frac{1}{1+z^n} - d_1 x$$

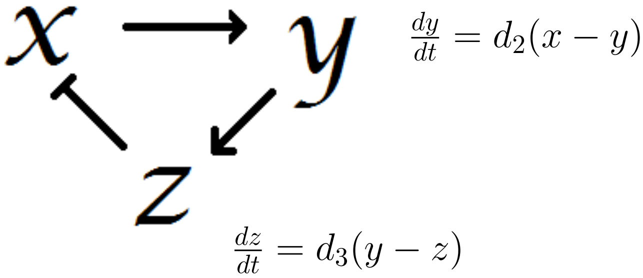

$$\frac{dy}{dt} = d_2(x - y)$$

$$\frac{dz}{dt} = d_3(y - z)$$

Both systems oscillate for sufficiently large Hill coefficients. Hill coefficients quantify the degree of cooperativity needed to obtain self-sustained oscillations.

500 different parameter sets were created and the corresponding critical m values (the lowest Hill coefficients which would still let the system oscillate) of the repressilator were plotted against the n values of the Goodwin oscillator. This was done by applying the Routh-Hurwitz criterion onto the Jacobian of each system to generate an inequality that has to be satisfied for limit cycle to occur. For each respective set of parameters, this inequality can be solved for the smallest possible Hill coefficient signalizing the transition from a stable to an unstable state.

Common parameters in both sets ($d1$, $d2$, $d3$ which are distributed by latin hypercube sampling (McKay et al., 2000) within a range from 0 to 1) have the same value, while $p2$ and $p3$ are chosen randomly for the

repressilator. As the combination of two high or two low p values might bias the cooperativity value, we assume $p2 \cdot p3 = 3$.

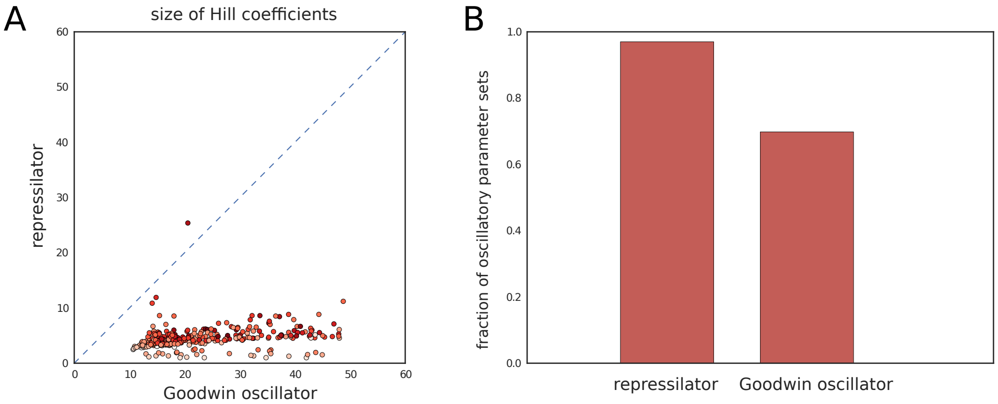

Figure S5-3: The repressilator is a more robust oscillator than the Goodwin oscillator (A) Minimal Hill coefficient producing oscillations in several parameter settings. Shown are 500 randomly chosen parameter sets represented as a point each. All parameters common to both models have the same value, except for the plotted Hill coefficient. The repressilator model needs in almost all cases lower values to produce oscillations. (B) Fraction of parameter sets that are oscillating per model for comparable parameter values.

Figure **S5-3**A shows the minimal n and m-values characterizing the necessary non-linearity of Goodwin model and repressilator, respectively. Consistent with the theory, the Hill coefficient n has to be larger than 8 (mean value $\bar{n} = 11.2$), whereas nonlinearities of the repressilator are smaller ($\bar{m} = 2.1$). The color intensity encodes the mean degradation rate. Note, that small degradation rates imply particularly small Hill-coefficients. We only considered systems with critical values $n \leq 50$ and $m \leq 50$. Figure **S5-3**B illustrates that almost all repressilators oscillate, whereas only about 70% of the Goodwin models exhibit oscillations for $n \leq 50$.

References

McKay, M. D., Beckman, R. J., and Conover, W. J. (2000). A comparison of three methods for selecting values of input variables in the analysis of output from a computer code. *Technometrics*, 42:55–61.
