# Supplementary material for: Feedback Loops of the Mammalian Circadian Clock Constitute Repressilator
Source: PLoS Comput Biol. 2016 Dec 12;12(12):e1005266. doi: 10.1371/journal.pcbi.1005266 (PMC5189953; doi:10.1371/journal.pcbi.1005266)
Supplement: S6 Appendix — mRNA expression phases in different tissues. (PDF) [file pcbi.1005266.s006.pdf]

S6 Phases of repressilator genes in mRNA expression

Motivation

To double-check the properties of the data-driven model, additional experimental data from a recently published study (Zhang et al., 2014) of different mouse tissues was analyzed. This data was used to check the potential compliance of the repressilator motif with different measurements and in various tissues.

Acquiring and fitting data

Affymetrix data of the genes constituting the repressilator motif was obtained for 13 tissues from the CircaDB database (`http://bioinf.itmat.upenn.edu/circa/`, dataset "mouse 1.0ST") and is also accessible at GEO under GSE54652. In 9 tissues the repressilator genes were found to be significantly rhythmic. A linear fit of trigonometric functions was conducted using "cosinor rhythmometry" (Nelson et al., 1979), assuming a fixed period of 24. Subsequently, the phase for each fitted function was determined analytically.

The measured mRNA expression at different time points could be resembled well by a simple sine-cosine-function. F-test p-values for all genes and tissues are ≤ 0.01. Thus, a simple shifted cosine function is regarded as a good representation of the data.

mRNA expression peak order is consistent with repressilator

The intrinsic mechanism generating oscillations is associated with a specific ordering of expression peaks. In case of the repressilator, peaks following the opposite direction of the inhibiting edges of the motif are expected, i.e. *Rev-erb-*$\alpha \rightsquigarrow$ *Per2* $\rightsquigarrow$ *Cry1*. Figure **S6-1** shows that while the phases vary between different tissues, the ordering is preserved within each individual tissue. This universal phase ordering is consistent with the repressilator motif.

References

Nelson, W., Tong, Y. L., Lee, J.-K., and Halberg, F. (1979). Methods for cosinor-rhythmometry. *Chronobiologia*, 6:305.

Zhang, R., Lahens, N. F., Ballance, H. I., Hughes, M. E., and Hogenesch, J. B. (2014). A circadian gene expression atlas in mammals: implications for biology and medicine. *Proc Natl Acad Sci U S A*, 111:16219–16224.

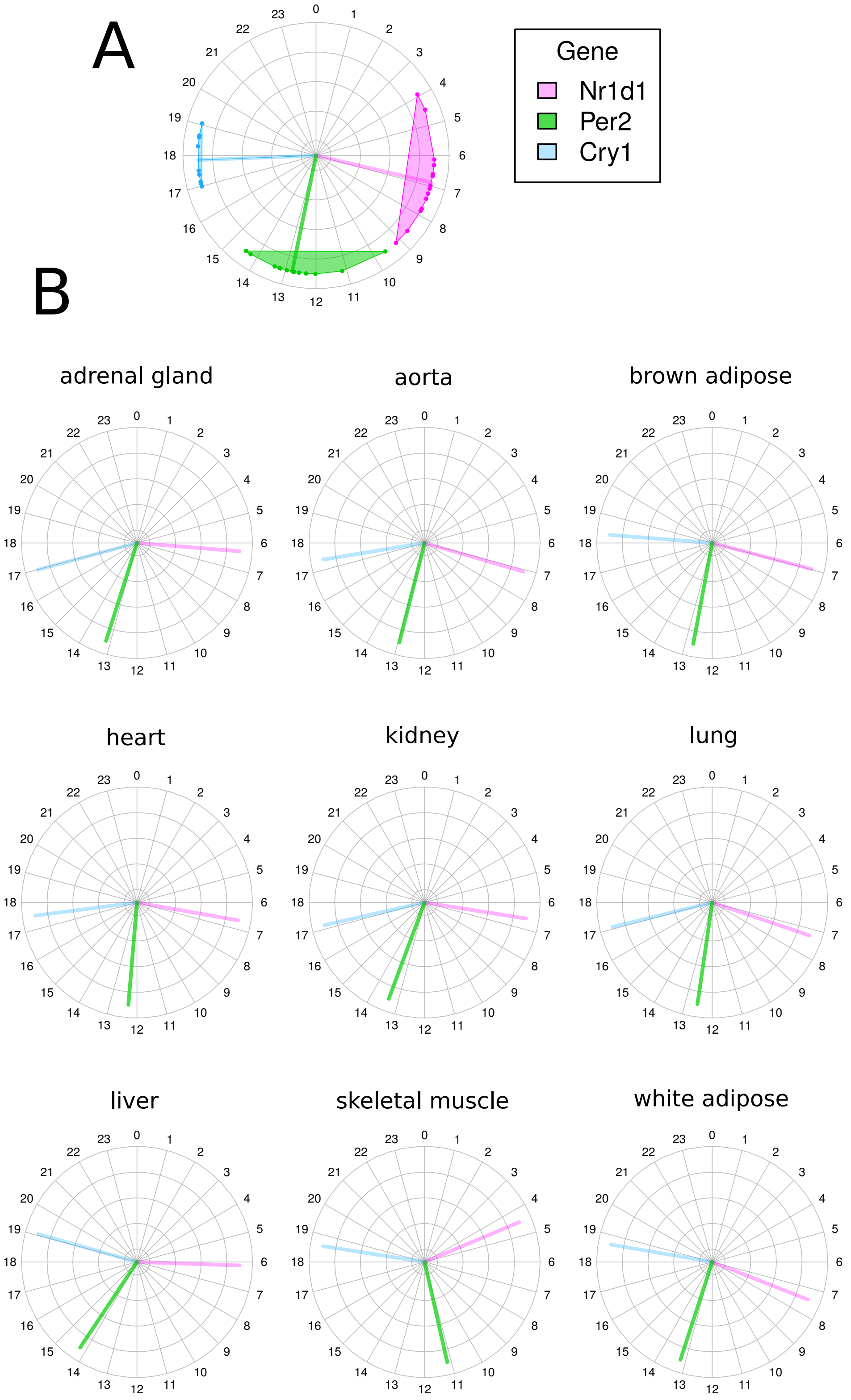

Figure S6-1: Phases determined for the three repressilator genes. (A) The distribution of each gene is shown as a colored polygon with a clockhand pointing to the mean value. (B) Phases shown separately for individual tissues. The order is consistent with the repressilator motif in all tissues.
