# Supplementary material for: Feedback Loops of the Mammalian Circadian Clock Constitute Repressilator
Source: PLoS Comput Biol. 2016 Dec 12;12(12):e1005266. doi: 10.1371/journal.pcbi.1005266 (PMC5189953; doi:10.1371/journal.pcbi.1005266)
Supplement: S7 Appendix — Repressilator in the same model structure fitted to kidney expression data. (PDF) [file pcbi.1005266.s007.pdf]

S7 Repressilator represents expression profiles in kidney

To assess the relevance of the repressilator motif in other tissues, we used parameter fits to experimental data taken from Zhang et al. (2014), using the same model formulation as in Korenčič et al. (2014). Parameters are derived using an optimization procedure described in a recent thesis (Kondoff (2015)).

Parameters, simulation and clamping

A timeseries of the kidney core clock model is shown in Figure **S7-1**A. We systematically clamped loops of the model and identified the same repressilator as in the network derived from liver and adrenal gland data to be essential for rhythmicity. Figure **S7-1**B and C illustrate the relevance of the repressilator: If we clamp one edge of the repressilator and interrupt this motif, oscillations vanish. However, if we clamp all other edges of the network except for the repressilator, rhythms with similar amplitude and phase as in the original model persist.

The parameters—which were used with the same model structure and equations as described in S1 Appendix—are listed in Table **S7-1**.

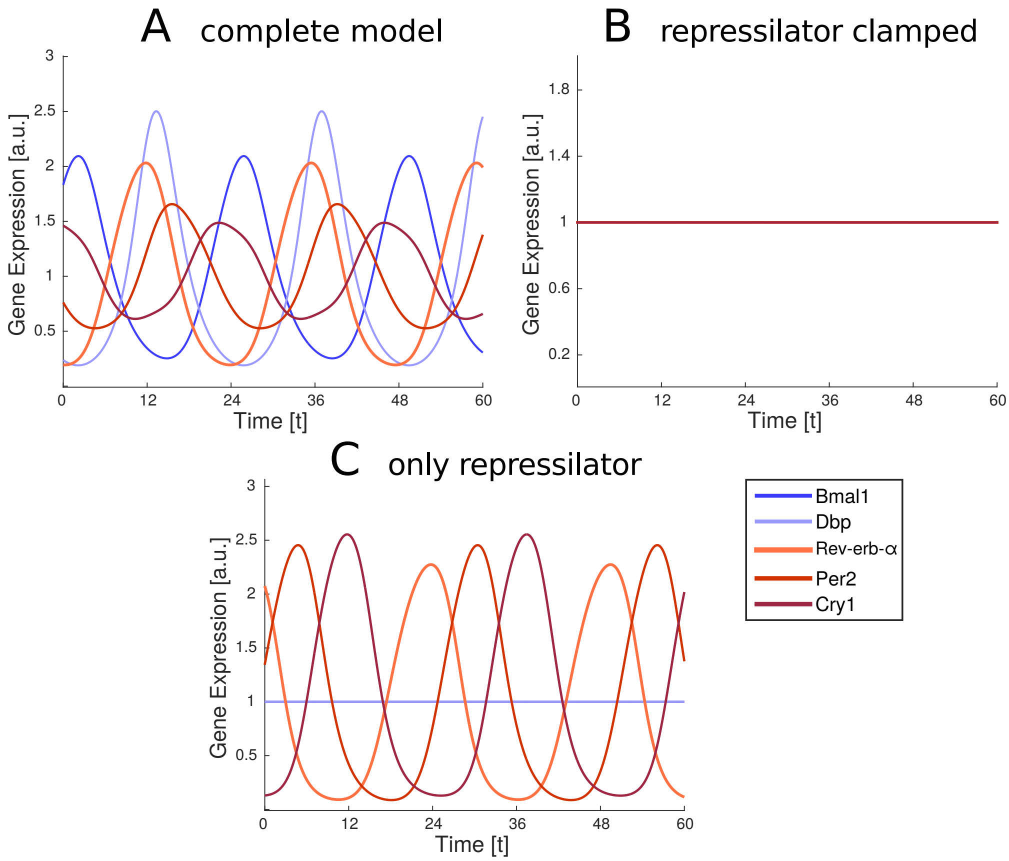

Figure S7-1: Simulation of a core clock model in mouse kidney under three conditions: (A) complete model, (B) one edge of the repressilator clamped (*Per2* ⊣ *Rev-erb-*α) and (C) all processes clamped, except for the repressilator edges. The plots highlight the essential role of the repressilator in this system.

τ_1	τ_2	τ_3	τ_4	τ_5	d_1	d_2	d_3	d_4	d_5
8.91	0.01	4.73	3.59	0.84	0.35	0.98	0.47	0.74	0.68

$inh_{2,1}$	$inh_{2,4}$	$inh_{3,2}$	$inh_{3,3}$	$inh_{3,4}$	$inh_{3,5}$	$inh_{4,2}$	$inh_{4,3}$	$inh_{4,4}$	$inh_{4,5}$
2.01	2.68	0.41	6.65	8.99	0.84	8.87	0.32	8.97	0.59

$actn_{1,2}$	$act_{1,2}$	$actn_{1,3}$	$act_{1,3}$	$actn_{1,4}$	$act_{1,4}$	$actn_{1,5}$	$act_{1,5}$	$act_{5,2}$
3.22	0.20	1.37	9.13	3.99	2.79	4.94	7.36	4.55

$actn_{5,2}$	$act_{5,3}$	$actn_{5,3}$	$act_{5,4}$	$actn_{5,4}$
1.69	6.30	2.21	0.68	3.86

Table S7-1: Parameter values of the kidney core clock model.

References

Kondoff, M. (2015). Modeling circadian clock gene networks in various tissues in mouse. Master's thesis, Charité Universitätsmedizin Berlin, Germany.

Korenčič, A., Košir, R., Bordyugov, G., Lehmann, R., Rozman, D., and Herzel, H. (2014). Timing of circadian genes in mammalian tissues. *Sci Rep*, 4:5782.

Zhang, R., Lahens, N. F., Ballance, H. I., Hughes, M. E., and Hogenesch, J. B. (2014). A circadian gene expression atlas in mammals: implications for biology and medicine. *Proc Natl Acad Sci U S A*, 111(45):16219–16224.
